# Reconciling Biodiversity Conservation and Widespread Deployment of Renewable Energy Technologies in the UK

**DOI:** 10.1371/journal.pone.0150956

**Published:** 2016-05-25

**Authors:** Benedict Gove, Leah J. Williams, Alison E. Beresford, Philippa Roddis, Colin Campbell, Emma Teuten, Rowena H. W. Langston, Richard B. Bradbury

**Affiliations:** 1 RSPB Centre for Conservation Science, Royal Society for the Protection of Birds, Sandy, Bedfordshire, SG19 2DL, United Kingdom; 2 RSPB Centre for Conservation Science, Royal Society for the Protection of Birds Scotland, Edinburgh, EH12 9DH, United Kingdom; 3 RSPB Sustainable Development Policy Team, Royal Society for the Protection of Birds, Sandy, Bedfordshire, SG19 2DL, United Kingdom; 4 RSPB Conservation Data Management, Royal Society for the Protection of Birds, Sandy, Bedfordshire, SG19 2DL, United Kingdom; 5 RSPB Centre for Conservation Science, The David Attenborough Building, Pembroke Street, Cambridge, CB2 3QZ, United Kingdom; Centro de Investigacion Cientifica y Educacion Superior de Ensenada, MEXICO

## Abstract

Renewable energy will potentially make an important contribution towards the dual aims of meeting carbon emission reduction targets and future energy demand. However, some technologies have considerable potential to impact on the biodiversity of the environments in which they are placed. In this study, an assessment was undertaken of the realistic deployment potential of a range of renewable energy technologies in the UK, considering constraints imposed by biodiversity conservation priorities. We focused on those energy sources that have the potential to make important energy contributions but which might conflict with biodiversity conservation objectives. These included field-scale solar, bioenergy crops, wind energy (both onshore and offshore), wave and tidal stream energy. The spatially-explicit analysis considered the potential opportunity available for each technology, at various levels of ecological risk. The resultant maps highlight the energy resource available, physical and policy constraints to deployment, and ecological sensitivity (based on the distribution of protected areas and sensitive species). If the technologies are restricted to areas which currently appear not to have significant ecological constraints, the total potential energy output from these energy sources was estimated to be in the region of 5,547 TWh/yr. This would be sufficient to meet projected energy demand in the UK, and help to achieve carbon reduction targets. However, we highlight two important caveats. First, further ecological monitoring and surveillance is required to improve understanding of wildlife distributions and therefore potential impacts of utilising these energy sources. This is likely to reduce the total energy available, especially at sea. Second, some of the technologies under investigation are currently not deployed commercially. Consequently this potential energy will only be available if continued effort is put into developing these energy sources/technologies, to enable realisation of their full potential.

## Introduction

The scientific case for climate change is well established [1], with widespread evidence indicating that terrestrial, freshwater and marine ecosystems are already being affected [2]. Furthermore, there is greater than 95% certainty that the dominant cause of climate change is the anthropogenic increase in greenhouse gas emissions [1]. Negative impacts on ecosystems, biodiversity, human health and food security are likely to escalate [2]. This presents a strong imperative to minimise future changes in climate by reducing anthropogenic greenhouse gas emissions. However, reducing emissions through use of renewable energy sources can also lead to environmental impacts [3]. Some impacts can be minimised through appropriate siting, design and innovation, but there may be difficult trade-offs between reducing emissions and conserving biodiversity. It is therefore essential to understand the ecological risks of the transition to a low carbon energy system.

Like other countries, the UK is committed to reducing biodiversity loss under agreements such as the Convention on Biological Diversity (including meeting the Aichi Biodiversity targets), the EU Biodiversity Strategy and the EU 2050 Vision [4]. In 2008, the UK became the first country to introduce a legally binding target to address climate change, set out in the Climate Change Act, 2008 [5]. The target is to achieve an 80% reduction in greenhouse gas emissions, relative to 1990 levels, by 2050 [5]. Meeting this ambitious target is likely to require improved energy efficiency, reduced demand for energy, and expanded deployment of renewable and low-carbon energy technologies. Using the UK as a case study, this paper aims to develop an approach for identifying the potential contribution to meeting national emissions targets of realistic renewable energy deployment scenarios that have minimal impacts on biodiversity.

A variety of renewable energy technologies are currently available commercially or are being developed. An assessment by BirdLife Europe [3] broadly categorised technologies as low, medium or high risk to biodiversity. Low risk technologies included rooftop solar, thermal and photovoltaic panels, and heat pumps (air or ground source). These are generally small-scale, involve little new infrastructure and do not result in major land-use changes. Risks to biodiversity from these technologies would be expected to be minimal and very localised [3].

Renewable technologies that were judged to have a high risk of negative impacts on biodiversity [3] include new large-scale hydro power schemes (e.g. those that create artificial reservoirs [6]) and certain tidal range technologies (particularly shore-to-shore barrages [3, 7]). It is possible that forms of these technologies might be developed that are more environmentally benign. However, at least for now, these technologies may be deployed only in very specific areas, after careful appraisal of their potential impacts under the Environmental Impact Assessment process.

Several technologies with medium risks to biodiversity [3] could be deployed widely and generate significant amounts of energy. Their deployment is likely to be crucial for meeting the target to reduce greenhouse gas emissions by 80% by 2050 [5]. These include onshore wind, bioenergy crops, solar farms, offshore wind (fixed base and floating turbines), wave power and tidal stream [3]. Each of these technologies has the potential to cause significant changes in land (or sea) use and therefore may affect the availability and suitability of habitats, as well as presenting a range of direct and/or indirect impacts on sensitive species. Tidal range energy was not included in this list as developments tend to occur in intertidal areas close to shore where the risk of impacts is generally expected to be high.

Given the potentially large energy contribution of these medium risk technologies, it is important to make a strategic assessment of their realistic deployment potential, taking into account constraints imposed by biodiversity conservation objectives. In this paper, a spatial analysis of potential deployment is presented for a range of medium risk renewable energy technologies. We consider the technical opportunity available for each technology and the constraints (physical, policy and ecological) that will limit deployment. As part of this process, we have used information and/or inferences about the effects of different renewable energy technologies on a range of species and habitats to produce technology-specific ecological sensitivity maps. This approach permits comparison of the overlap of potential areas for the deployment of specific renewable energy technologies with species or habitats that may be negatively impacted by their presence. It also enables the estimation of the total energy-generation potential, at high, medium and low overall ecological risk, of these medium risk renewable technologies within the UK.

Two scenarios are explored in detail in this paper. We identify a *low ecological risk scenario* that excludes areas of both high and medium ecological sensitivity from the areas of energy-generating opportunity remaining after physical and policy constraints have been applied. This scenario represents the areas that are estimated to be available for the potential deployment of renewable energy technologies without posing significant risks to biodiversity of conservation concern. We also identify a *medium ecological risk scenario* that excludes only areas of high ecological sensitivity from the areas of opportunity remaining after the application of physical and policy constraints. This represents the areas estimated to be available for deployment where some biodiversity impacts might occur, depending on the scale of deployment, the location of specific sites and the extent to which site-specific mitigation is utilised. Finally, we were able to consider the potential contribution of the renewable energy technologies under consideration towards meeting carbon emissions reduction targets. This study is not intended as a full environmental impact assessment of whole energy systems, but specifically of those renewable technologies that have the potential to impact on conservation objectives but will also play an important role in the generation of relatively cheap low carbon energy in the UK, in the foreseeable future.

## Materials and Methods

Our approach was based on that of SQWenergy [8]. This spatial approach is sequential; firstly the available resource (or opportunity) of each energy source is mapped, before accounting for physical and policy constraints to the deployment of technologies that can harness the resource. Physical constraints reflect restrictions to the utilisation of the energy source that are unlikely to change in the foreseeable future, such as the presence of incompatible infrastructure (e.g. roads and railways), or where the landscape is unsuitable for deployment. Policy constraints reflect a range of policy, planning and regulatory restrictions. They can be more flexible than physical constraints and could change with shifts in policies and/or public opinion. Together, these constraints reduce the total from theoretical opportunity to that which is potentially achievable, in other words the ‘practical opportunity’.

We take this is our starting point and extend the approach to incorporate ecological sensitivity as a further potential constraint to the deployment of renewable energy technologies. The last mapping step is therefore to identify the residual areas of opportunity that have low and medium ecological sensitivity.

Once all constraints have been incorporated, the potential *installed capacity* for the area remaining was then estimated by multiplying the area by the estimated average power density per unit area for that technology, to give the potential [9]. The annual energy output was then calculated as follows:

Annual energy output (TWh/yr)= Installed capacity (TW)x 8,765.81(hours in a year)x Load factor (1)

Where the *load factor* is the amount of energy produced over a period of time by a technology, expressed as a percentage of its rated potential output, were it producing energy at full capacity continuously over that time period.

All mapping was carried out in ArcGIS version 10.1 [10]. All maps were projected to the British National Grid (BNG) and converted to a 1 km^2^ resolution. Each 1 km^2^ square was assigned a value within each layer (opportunity, physical constraint, policy constraint and ecological sensitivity) if more than 50% of the area was characterised by that value. A ‘point and buffer’ approach was followed throughout the mapping process, whereby buffer zones were applied, where appropriate, to define an additional zone of constraint beyond the boundary of a feature. For example, buffers are applied alongside motorways for safety reasons, where 165m is applied to wind turbines whereas 15m is used for solar/bioenergy crops. Buffer zones are also an established feature of ecological sensitivity mapping [11].

### Opportunity Mapping

The available resource for extracting energy from onshore wind depends primarily on wind speed and the technological ability to harness the energy, in terms of turbine size, efficiency and installation requirements. Wind speed data for the UK were downloaded from the NOABL (Numerical Objective Analysis Boundary Layer) wind speed database [12], which maps annual average wind speed at 10 m, 25 m and 45 m above ground level, at a resolution of 1 km^2^. All areas with average wind speeds of at least 5 m/s at 45 m above ground level were considered potentially suitable for commercial wind development.

Opportunity mapping for dedicated energy crops was largely based on SQWenergy methods [8], supplemented by information from Lovett et al [13], which mapped land suitable for *Miscanthus* planting. In order to assess maximum potential it was assumed that all areas of arable land and pasture are capable of growing energy crops. We used Land Cover Map (LCM) 2000 vector data [14] at 25m resolution (supplied by the Centre for Ecology and Hydrology, CEH) and selected all areas classified as arable cereals, arable horticulture, non-rotational horticulture, improved grassland or set-aside grass (i.e. all land cover types in categories 4 and 5 in LCM2000). However, not all areas identified in the mapping for bioenergy crops will be suitable for their cultivation. For example, large parts of the southwest of England may not have the correct climate, soil or water availability for successful production [15]. There may be similar issues for solar farms.

There is no set threshold, in terms of irradiance or energy output, below which commercial solar panels are considered non-viable. At present, siting depends on a balance between irradiance and economic considerations, such as the cost of connecting to the grid or land rental prices. As economic incentives could change significantly between now and 2050, and the technology is being continually improved, we consider all areas as potentially viable. However, irradiance (and therefore potential energy output), does vary across the UK. We therefore downloaded a solar radiation map, from the European Commission’s Institute for Energy and Transport [16], which mapped the average yearly sum of global irradiation on an optimally inclined (static) surface (in kW/m^2^). These data formed part of the original PVGIS (Photovoltaic GIS) solar radiation data set, based on ground station data from 1981–1990 and were mapped at a resolution of 1 arc-minute. The map was re-projected to BNG and re-sampled to a resolution of 1 km^2^. It was assumed for the purposes of this study that floating solar on inland water-bodies will not be deployed to the extent that it was worth including this technology in the analysis. The physical and policy constraints mapped for ***onshore technologies*** are listed in S1 and S2 Tables.

For offshore technologies we used opportunity maps made available by The Crown Estate for offshore wind (fixed and floating turbines), wave and tidal stream energy, from their Marine Resources System (MaRS) database. The maps were created by modelling variation in suitability across the seascape, as a function of a specific set of technical parameters for each technology (S3 Table), weighting each parameter according to the standard procedure employed by The Crown Estate in their resource assessment process. It should be stressed that these maps vary in confidence and given the stage that they were used, they are indicative of future potential at a high level only, to assist with presenting a strategic national overview. The model outputs were normalised, then split into three equal bands (0–0.33/0.33–0.66/0.66–1) to distinguish prime, good and technical opportunity. These categories are defined in the following way:

*Prime opportunity*–locations where the deployment of renewable technology is likely to deliver energy at lower/optimal cost.
*Good opportunity*–areas where the deployment of renewable technologies is likely to be commercially viable, in line with predicted market conditions.
*Technical opportunity*–areas where appropriate physical conditions exist to support the deployment of devices using available renewable technologies, but development is less economically viable.

This banding was designed to help ensure that the outputs realistically indicated deployment opportunity. Technical opportunity exists over much of UK waters (especially for floating technologies), but it is unrealistic to assume a large deployment in remote areas, far from centres of demand, where the costs of deployment, connection with the power grid and maintenance will be high.

Fixed-base turbines currently can be deployed in sea depths up to 60 m, whereas floating turbines can be deployed in sea depths of 50 m or more. To account for this overlap, the 50–60 m depth contour was removed from the floating wind opportunity layer. Suitability for wave and tidal stream technologies was modelled based on parameters provided by existing technology developers, as these technologies are still in the development phase. Tidal stream and wave energy technologies are currently under development, with only a few small-scale commercial deployments, such as Meygen in the Pentland Firth and SeaGen in Strangford Lough. Several other developments are in the planning or development phases in UK waters. To date, developers have relied on underwater turbine technologies, but many novel designs have been proposed and some are currently being tested.

Physical and policy constraints for offshore technologies were obtained from the MaRS database. Physical constraints were areas with existing infrastructure or alternative seabed use (S4 Table). Our analysis assumes these constraints will remain in place until 2050, although some seabed agreements may expire and infrastructure may be decommissioned in this timeframe. However, new sites are likely to be commissioned in their place in many cases. Policy constraints to offshore renewable technologies are presented in S5 Table. Due to the complexities in the use of offshore environments, a more nuanced approach was necessary, so the policy constraints were split into three categories. Policy constraint level one is the least constrained and level three is the most constrained. Where more than one policy constraint overlapped, the maximum value was taken. We have presented the results in a matrix form that allows examination of the trade-offs between different constraints to deployment.

### Ecological sensitivity mapping

**Fig 1:**
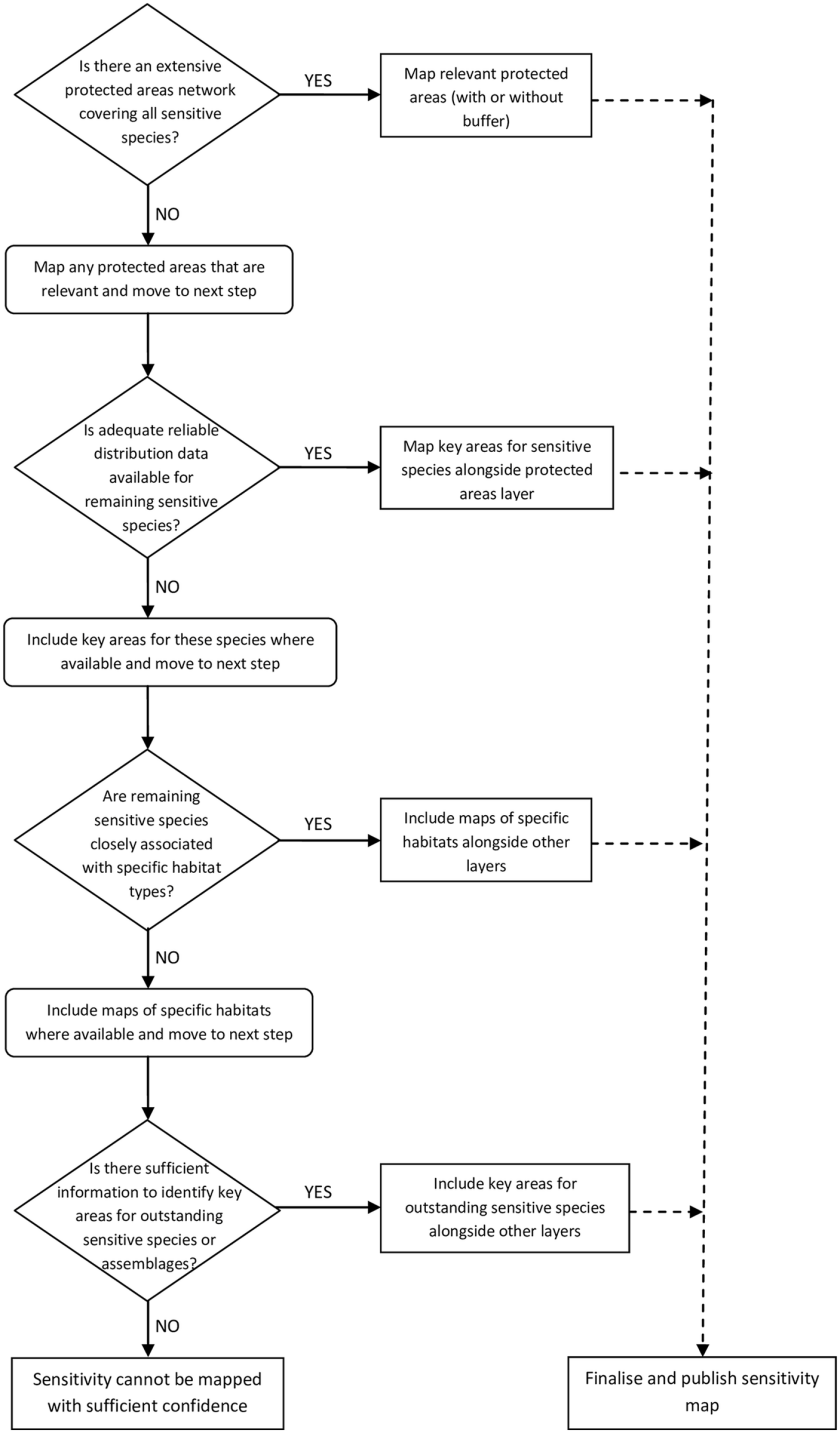
Ecological sensitivity mapping decision tree.

Ecological sensitivity maps have been used previously to show the distribution of species and habitats that are sensitive to specific technologies, e.g. [11, 17, 18, 19]. However, there has been a lack of comprehensive ecological sensitivity mapping for any technology apart from onshore wind energy in the UK or its constituent countries, and there is considerable uncertainty surrounding the nature and extent of impacts. Therefore, we also incorporated a range of habitat types and designated sites, to account for potential impacts such as habitat loss and disturbance to avian and non-avian taxa. We produced UK-wide sensitivity maps for bioenergy crops and solar farms, onshore and offshore wind, wave and tidal stream energy technologies, incorporating designated sites and sensitive habitats, and including a species-specific approach that focussed on bird species and assemblages. A decision tree was used to determine the mapping approach for each technology (Fig 1).

#### Site-based ecological sensitivity mapping

In the UK, areas protected under national or international law for their ecological features are likely to correspond closely to the distribution of rare or threatened species, or congregations of migratory species [20, 21, 22]. These species are most likely to be sensitive to impacts arising either directly from development (including renewable technologies), or from the associated loss of habitat or land-use change [3]. As a consequence, the starting point for our ecological sensitivity maps was the distribution of protected sites (S6 Table). Buffers were not applied to protected areas because (i) sensitivities of qualifying species to energy projects are often unknown and/or species-specific; (ii) protected area boundaries often have integral buffers; and (iii) because of the risk of over-estimating areas of sensitivity. We considered sites protected by international and national legislation to be high sensitivity, with all other designations treated as medium sensitivity. Certain habitat types were allocated a medium sensitivity score, as potentially sensitive species associated with them could be negatively affected by land use change associated with certain renewable technologies (S6 Table). Deep peat was ascribed medium sensitivity to onshore wind due to the risk of slumps/landslides during construction activities, although we recognise that wind farms can be accommodated in some peatland areas if well micro-sited and commitments are made to peatland restoration/enhancement. Ancient semi-natural woodland was allocated medium sensitivity to onshore wind as it is a rare habitat of conservation priority. Semi natural grassland provides habitat for important and rare species, including many of those of conservation concern, and thus was allocated medium sensitivity to bioenergy crops and solar farms. Organic and peat soils were considered unsuitable for the cultivation of energy crops [13] owing to their importance for species of conservation concern and for carbon storage/sequestration and were allocated medium sensitivity to bioenergy crops and solar farms.

The designated sites network offshore is not as well developed as it is onshore. Therefore, as well as established designated sites, we included several areas that qualify as marine Special Protection Areas for seabirds [23]. Offshore Important Bird Areas (IBAs) [24] were given a high sensitivity rating. National Nature Reserves (NNRs) were allocated medium sensitivity for offshore renewables only, as onshore NNRs are already covered by the international/national designated sites network.

#### Species-based ecological sensitivity mapping

Protected species are highly variable in the proportions of their populations contained within protected areas, with dispersed species (particularly those in upland areas) poorly represented [25]. Therefore, avoidance of deployment at protected sites cannot be assumed adequate to avoid all impacts on sensitive species. Consequently, each sensitivity map includes the distribution of those species believed to be sensitive to a particular renewable technology, in addition to the designated sites network. Since data on the distribution of some sensitive species is rarely sufficiently comprehensive to prove their absence it is not usually possible to distinguish between areas of low sensitivity and those where the sensitivity is unknown, this is acknowledged in the mapping process by referring to these areas as low/unknown sensitivity. This is particularly true for offshore areas.

The methods for the onshore wind sensitivity mapping repeated those for the avian sensitivity maps for England and Scotland (see [11, 17, 18] for detailed methods and limitations to the approach), using a species list combined from both sources and adding additional available species distribution data (collected between 2009 and 2012). Species included (i) those where the literature indicated sensitivity to collision risk, disturbance or changes in habitat; and (ii) species undergoing rapid population declines or with very localised populations [11, 18]. Details of species, data sources, buffer distances and sensitivity levels are given in S7 Table. Older datasets of species distributions were allocated a medium sensitivity score, as data may not reflect recent changes in distribution or abundance.

The impacts of wide-scale planting of energy crops or the deployment of solar farms on biodiversity in the UK are not well known. Whereas studies of pioneer crops have shown largely positive responses e.g. [26, 27, 28], no landscape-scale empirical studies have been conducted on the subject as these features are not currently widely deployed. Therefore, the sensitivity mapping for these technologies focused on bird species that would be likely to be negatively impacted by land use change and intensification of field management *per se*, rather than energy crops specifically. One application of species-based sensitivity mapping was used for both technologies.

Species considered potentially sensitive to these technologies were those that are sensitive to the loss of seed rich habitats or semi-natural grassland and species closely associated with wet grassland or open upland habitats. Some of these species are the subjects of regular monitoring programmes or national surveys and sufficient information was available to include them at species level in the sensitivity mapping process (S8 Table). For these species, the same ‘point and buffer’ approach was taken as for the onshore wind sensitivity mapping [11], with high and medium sensitivity buffers drawn around breeding locations or aggregations recorded in national surveys and other monitoring schemes. Buffer distances, where known, were based on literature reviews of disturbance/displacement distances and foraging ranges.

Many of the species identified as being potentially sensitive to energy crop planting or solar farm deployment are not the subject of specific monitoring schemes. For these species, the best available data (presence/absence data at a 10 km^2^ resolution) were from the most recent BTO Bird Atlas, 2007–2011, which is a joint project between BTO, BirdWatch Ireland and the Scottish Ornithologists’ Club [29]. It was considered that an assemblage approach would make best use of such coarse resolution data, with all 10 km^2^ squares with certain combinations of species triggering qualification as medium sensitivity. Species were assigned to one of three assemblages: arable, farmland waders or upland waders (S9 Table). Each assemblage was mapped separately. Instead of assessing species richness in each 10 km^2^, which was strongly influenced by widespread species, we used a ‘diversity index’ to weight the conservation interest of these species. Each species was given a score of (1/number of 10 km^2^ squares in the UK in which it was present), such that species with more restricted distributions were assigned higher weight than those with more widespread distributions. Scores were then summed across all species in the assemblage, and squares scoring in the top quartile (25%) were classified as medium sensitivity.

The sensitivity mapping for offshore species focused on breeding seabirds, supplemented by at-sea distributions of wintering seabirds [30] and marine megafauna [31]. We focused on breeding UK seabird species listed as potentially sensitive by Furness et al. [32, 33] for which colony counts were undertaken as part of the Seabird 2000 census. Each colony was buffered by the mean maximum foraging distance for the species [34] (S10 Table). The area of sea within each buffer was calculated, along with the associated density of birds, assuming the birds from each colony to be uniformly distributed throughout that colony’s buffer e.g. [35]. This is unlikely to be a true reflection of a species’ distribution, but in the absence of detailed information on habitat quality and foraging associations this approach was assumed to be the most precautionary. An estimated density map was created for each species by mapping colony buffers (with their associated density values) and summing density values where buffers overlapped. To account for differences in species’ sensitivity to renewable technologies, we used the species sensitivity scores (SSS) from Furness et al. [32, 33] as weighting factors, following closely the method outlined in Bradbury et al. [30].

This resulted in four mapped sensitivity layers on continuous scales. For consistency with our onshore sensitivity mapping approach, we then applied thresholds to categorise each layer into high sensitivity, medium sensitivity and low/unknown sensitivity areas. These thresholds were set using Jenks natural breaks optimisation [36]. This method seeks natural clustering in the data by reducing the variance within classes whilst maximising the variance between classes (S11 Table). For offshore wind, separate thresholds were applied to the collision and disturbance/displacement maps and the results combined, selecting the maximum sensitivity category available in each grid square for the sensitivity map.

For offshore wind technologies, species-based offshore sensitivity mapping was supplemented with data layers from SeaMaST (Seabird Mapping and Sensitivity Tool) [30], to account for the distribution of overwintering seabirds and those that do not breed in the UK. This freely available tool utilises information from the European Seabirds at Sea (ESAS) database and consists of boat-based and aerial survey data collected between 1979 and 2012. The ‘Winter Combined’ map, consisting of distribution data on wintering seabirds at risk of collision and/or displacement by offshore wind turbines, was added as a layer to the offshore wind sensitivity map (the two datasets were combined using the highest ranking score for each species from either risk). For full methods see Bradbury et al [30]. For consistency, the scores were split into three categories; the top two categories were allocated high sensitivity (scores of 57–98) and medium sensitivity (scores of 32–57), respectively, whereas all lower scores (0–31) were allocated a low/unknown sensitivity rating.

In order to account for other marine wildlife in our analysis, we supplemented our data with the Additional Pelagic Ecological Importance (APEI) data layer [31]. This data layer provides information on areas of pelagic biodiversity around the UK, including data on thermal fronts, Whale and Dolphin Conservation (WDC) important areas for marine mammals, Cefas and ICES nursery and spawning data based on plankton surveys and Marine Conservation Society and Shark Trust basking shark sightings (see S12 Table). However, the layer is unlikely to represent a complete inventory of important areas due to significant data gaps. Within this layer, the Additional Ecological Information (AEI) score for a particular grid cell is calculated by summing the score from each dataset. The seabird foraging radii layer was removed, as this was already accounted for in our analysis, and the AEI scores recalculated. Jenks natural breaks optimisation [36] was again used, to split the dataset into three. Only the top scores were used (an AEI score of 5 or above) and were allocated a medium sensitivity rating. Medium sensitivity was selected because the layer represents a density surface of marine species abundance, rather than sensitivity to a particular technology.

To produce the final sensitivity maps for each technology, a composite map was created from the species and assemblage distribution layers and then added to the protected sites layer, see Eqs 2–6. If more than one sensitivity value was allocated to a single 1 km^2^ cell, the highest sensitivity value was selected.

$$\text{Onshore wind sensitivity index }\left(\text{SI}\right)=\text{max}\;\left\{Protected\;areas\;score,\;\;Ancient\;semi-natural\;woodland\;score,\;Bog\;score,\;Species_{1}\;score,\;\ldots ,\;Species_{n}\;score\right\}$$ (2)

Where:

Protected areas score=low; medium; high

Habitat scores (Ancient semi-natural woodland;Bog)=low; medium

$${Species\;scores}_{1-n}=\text{low; medium; high}$$

$$\begin{array}{l} \text{Solar/biomass SI}=\\ \text{max }\{Protected\;areas\;score,\;Semi-natural\;grassland\;score,\;Organic\;and\;peat\;soils\;score,\;Arable\;birds\;assemblage\;score,\;Farmland\;waders\;assemblage\;score,\;Upland\;waders\;assemblage\;score,\;Species_{1}\;score,\;\ldots ,\;Species_{n}\;score\} \end{array}$$ (3)

Where:

Protected areas score=low; medium; high

Habitat scores (Semi-natural grassland; Organic and peat soils)=low; medium; high

Assemblage scores (Arable; Farmland; Upland)=low; medium [top 25%]

$${Species\;scores}_{1-n}=\text{low; medium; high}$$

Offshore wind SI=max{Protected areas score,  Collision sensitivity score, Displacement sensitivity score, Wintering seabird score, APEI score} (4)

Where:

Protected areas score=low; medium; high

$$Seabird\;collision\;sensitivity\;score\;=\;\sum_{species}\left\{\text{ln}(density_{species}+1)\;\times SSS_{collision}\}=\text{low }[<739];\;medium\;[739-2,346];\;high\;[>2,346\right]$$

$$Seabird\;displacement\;sensitivity\;score\;=\;\sum_{species}\left\{\text{ln}(density_{species}+1)\;\times SSS_{displacement}\}=\text{low }[<34];\;medium\;[34-92];\;high\;[>92\right]$$

$$Wintering\;seabird\;score\;=\;\sum_{species(winter)}\left\{\text{ln}(density_{species(winter)}+1)\;\times SSS_{\text{max}(collision,\;\;\;displacement)}\}=\text{low }[<32];\;medium\;[32-56];\;high\;[>56\right]$$

APEI score=(Basking shark score [0−3]+ Marine mammal score [0−3]+ Nursery and spawning grounds score [0−3]+ Thermal front score [0−3])= low [0−4]; medium [>4]

Tidal SI=max {Protected areas score,  Tidal sensitivity score, APEI score} (5)

Where:

Protected areas score=low; medium; high

$$Tidal\;sensitivity\;score\;=\;\sum_{species}\left\{\text{ln}(density_{species}+1)\;\times SSS_{tidal\;stream}\}=\text{low }[<4];\;medium\;[4-8];\;high\;[>8\right]$$

APEI score=(Basking shark score [0−3]+ Marine mammal score [0−3]+ Nursery and spawning grounds score [0−3]+ Thermal front score [0−3])= low [0−4]; medium [>4]

Wave SI=max {Protected areas score, Wave sensitivity score, APEI score} (6)

Where:

Protected areas score=low; medium; high

$$Wave\;sensitivity\;score\;=\;\sum_{species}\left\{\text{ln}(density_{species}+1)\;\times SSS_{wave}\}=\text{low }[<440];\;medium\;[440-1,461];\;high\;[>1,461\right]$$

APEI score=(Basking shark score [0−3]+ Marine mammal score [0−3]+ Nursery and spawning grounds score [0−3]+ Thermal front score [0−3])= low [0−4]; medium [>4]

## Results

### Onshore wind

**Fig 2:**
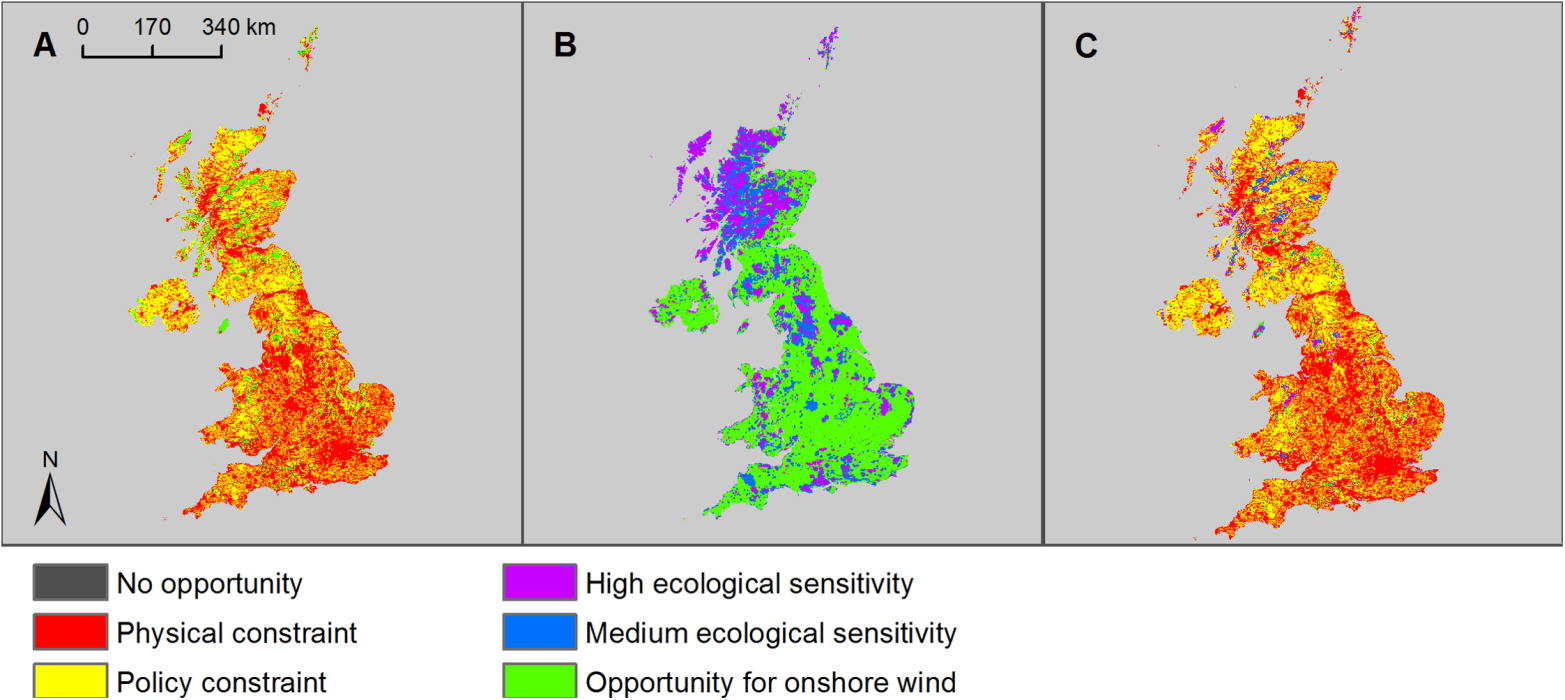
Opportunity and constraint mapping for onshore wind energy. A) Opportunity map for commercial-scale onshore wind development in the UK (green areas) showing physical constraints (red areas) and policy constraints (yellow areas); B) ecological sensitivity map for onshore wind energy showing high sensitivity (purple areas), medium sensitivity (blue areas) and low/unknown sensitivity (green areas); and C) composite map showing remaining areas of opportunity with low/unknown ecological sensitivity after all constraints have been applied (green areas).

**Fig 3:**
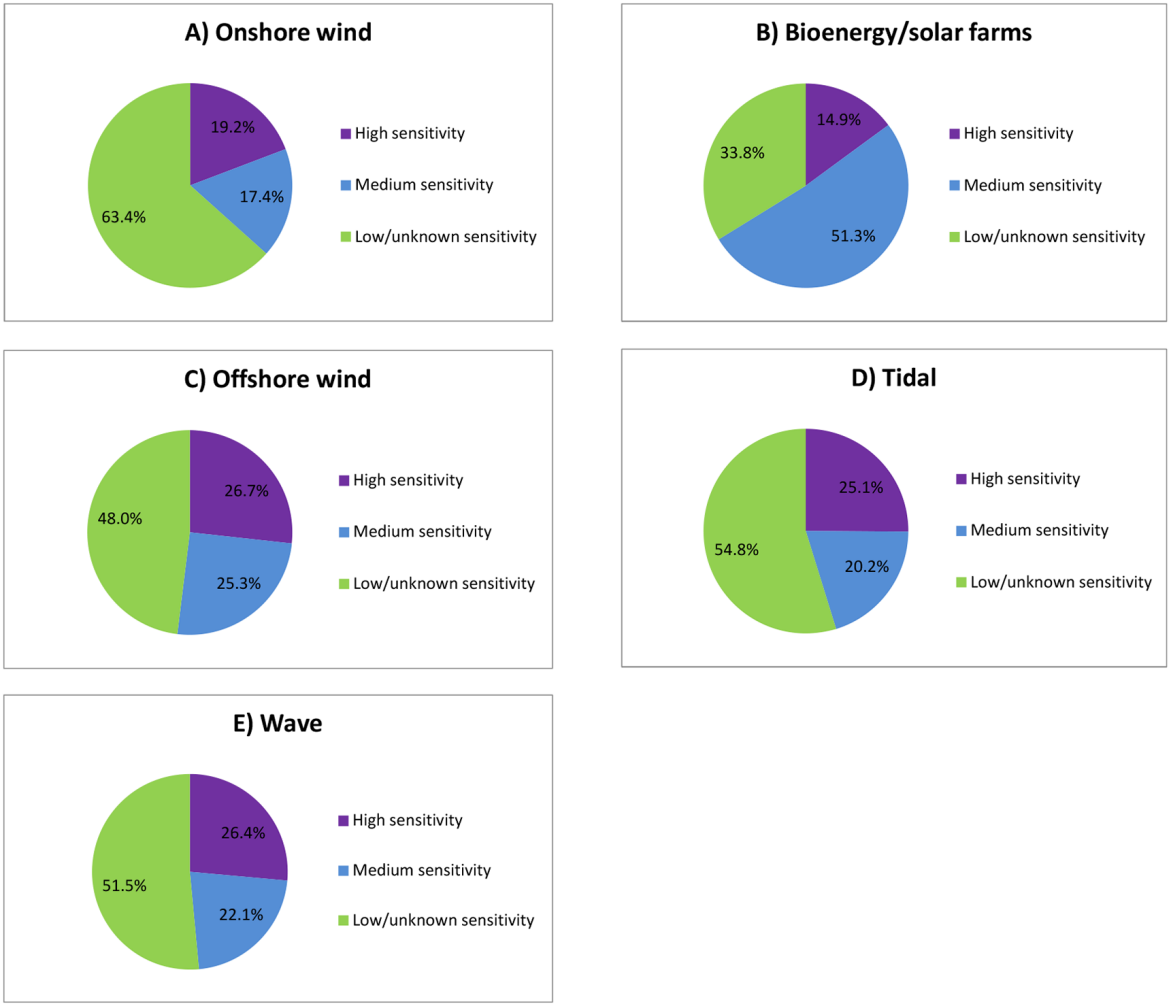
Estimated proportions of the UK with different ecological sensitivities to ‘medium risk’ renewable technologies. A) Proportion of UK land area of low/unknown, medium and high sensitivity to onshore wind energy; B) proportion of UK land area of low/unknown, medium and high sensitivity to bioenergy or solar farms; C) proportion of UK sea area of low/unknown, medium and high sensitivity to offshore wind energy; D) proportion of UK sea area of low/unknown, medium and high sensitivity to tidal energy; and E) proportion of UK sea area of low/unknown, medium and high sensitivity to wave energy technologies.

The majority of the UK (96.3% of the total land area) has sufficient wind resource to be suitable for harnessing wind energy (S13 Table). The application of physical and policy constraints reduced this potential to 6.9% of UK land area—around 16,976 km^2^ (Fig 2A, Table 1). In total, 19.2% of the UK land area had high ecological sensitivity to wind turbines and 17.4% had medium ecological sensitivity, leaving 63.4% as low/unknown sensitivity (Figs 2B and 3). In the low ecological risk scenario 5,932 km^2^ (2.4% of the UK land area) was potentially available for onshore wind deployment (Fig 2C, Table 1). In the medium ecological risk scenario 10,523 km^2^ (4.3% of the UK land area) was potentially available for onshore wind energy generation.

**Table 1:** Estimated energy available through the deployment of onshore renewable energy technologies in the UK at low, medium and high risk of ecological impacts. Land areas available for the deployment of commercial-scale onshore wind, solar and biomass energy; potential installed capacity and annual energy outputs considering the available resource after physical and policy constraints are applied.

Renewable energy technology	High ecological risk			Medium ecological risk			Low ecological risk		
	(no sensitivity applied)			(high sensitivity areas excluded)			(high and medium sensitivity areas excluded)		
	Area (km^2^) [relative to UK land area]	Potential installed capacity (GW)	Annual energy output (TWh/yr)	Area (km^2^) [relative to UK land area]	Potential installed capacity (GW)	Annual energy output (TWh/yr)	Area (km^2^) [relative to UK land area]	Potential installed capacity (GW)	Annual energy output (TWh/yr)
**Onshore wind**^a^	16,976 [6.9%]	153	402	10,523 [4.3%]	95	249	5,932 [2.4%]	53	140
**Solar**^b^	146,028 [59.8%]	3,213	2,535	134,793 [55.2%]	2,965	2,340	56,808 [23.3%]	1,250	986
**Biomass**^c^	104,289 [42.7%]	78	362	102,058 [41.8%]	77	355	46,138 [18.9%]	35	160

^a^ Onshore wind: power density = 9 MW/km^2^ [8]; load factor = 0.30 [37, 38].

^b^ Solar photovoltaics: power density = 22 MW/km^2^ [9]; load factor = 0.09 [37, 38].

^c^ Biomass electricity: power density = 0.75 MW/km^2^ [9]; load factor = 0.53 [37, 38].

An average power density of 9 MW per km^2^ was used for the purpose of this analysis [9]. A load factor of 0.30 was used, which was the mean for onshore wind turbines in the UK for 2009–2014, based on average beginning and end of year capacity [37, 38] and assuming a modest improvement in the technology over the coming years. The resultant potential annual energy output for onshore wind was 140 TWh/yr for the low ecological risk scenario and 249TWh/yr for the medium ecological risk scenario (Table 1).

### Bioenergy crops

**Fig 4:**
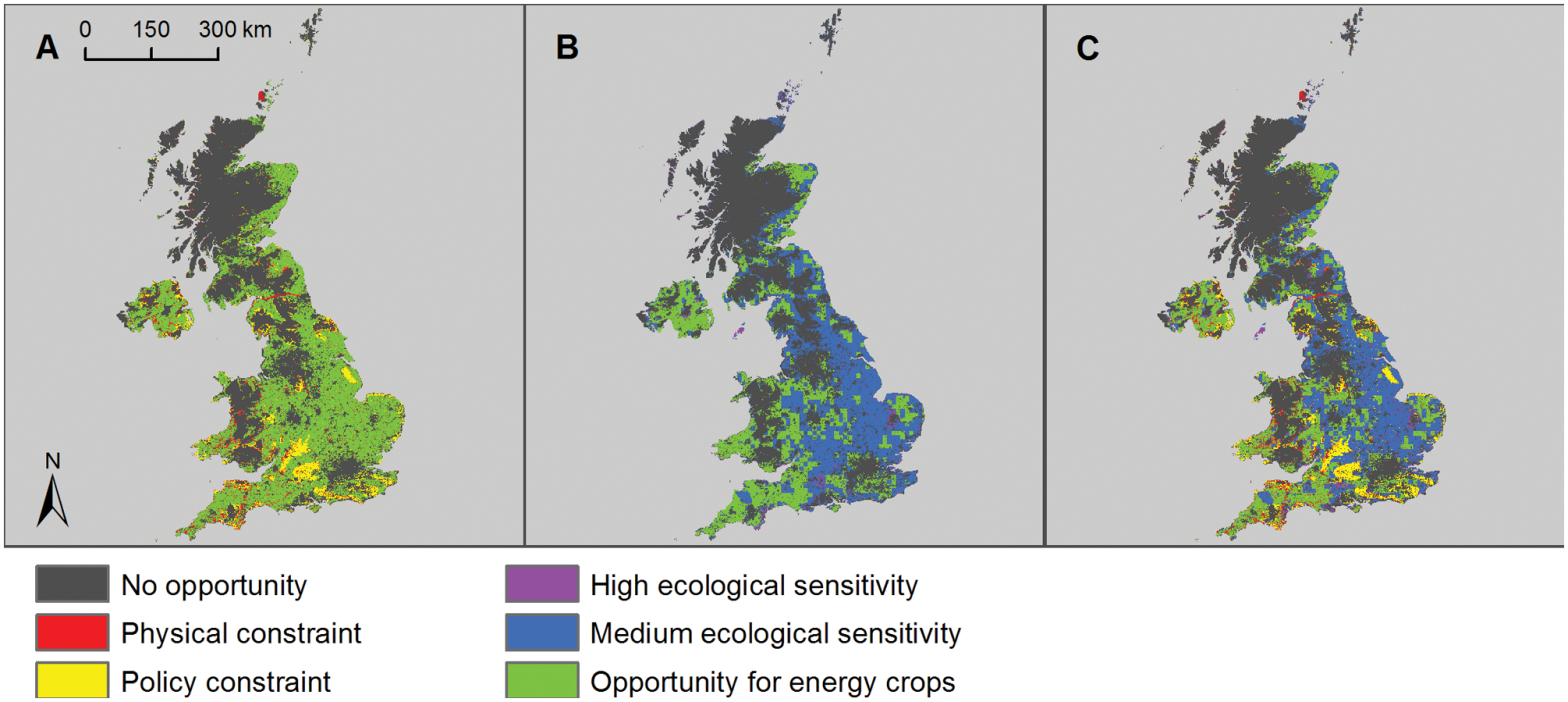
Opportunity and constraint mapping for bioenergy crops. A) Opportunity map for bioenergy crops in the UK (green areas) showing physical constraints (red areas) and policy constraints (yellow areas); B) ecological sensitivity map for bioenergy showing high sensitivity (purple areas), medium sensitivity (blue areas) and low/unknown sensitivity (green areas); and C) composite map showing remaining areas of opportunity for bioenergy crops with low/unknown ecological sensitivity after all constraints have been applied (green areas).

Opportunity mapping showed that 52.4% of the UK was technically suitable for growing energy crops (S13 Table). The addition of physical and policy constraints reduced this potential to 42.7% of UK land area (Fig 4A, Table 1). A total of 14.9% of the UK land area had high ecological sensitivity and 51.3% had medium ecological sensitivity to energy crop cultivation or solar farm deployment, leaving 33.8% as low/unknown sensitivity (Figs 3 and 4B). In the low ecological risk scenario 46,138 km^2^ (18.9% of the UK land area) was available for the cultivation of energy crops (Fig 4C, Table 1). Under the medium ecological risk scenario 102,058 km^2^ (41.8% of the UK land area) was left available for energy crops.

An average power density for bioenergy crops of 0.75 MW/km^2^ was used in this analysis, representing the best performance of *Miscanthus* grown in the UK [13]. A load factor of 0.53 was used, being the mean value for bioenergy from plant biomass in the UK between 2010 and 2014, based on beginning and end of year capacity [37, 38]. The resultant annual energy output for energy crops was 160 TWh/yr for the low ecological risk scenario and 355 TWh/yr for the medium ecological risk scenario (Table 1). If the heat from combustion of energy crops is also utilised these figures potentially become 303 TWh/yr and 671 TWh/yr, for the low and medium risk scenarios respectively (assuming that all of the embedded energy can be utilised).

### Solar farms

**Fig 5:**
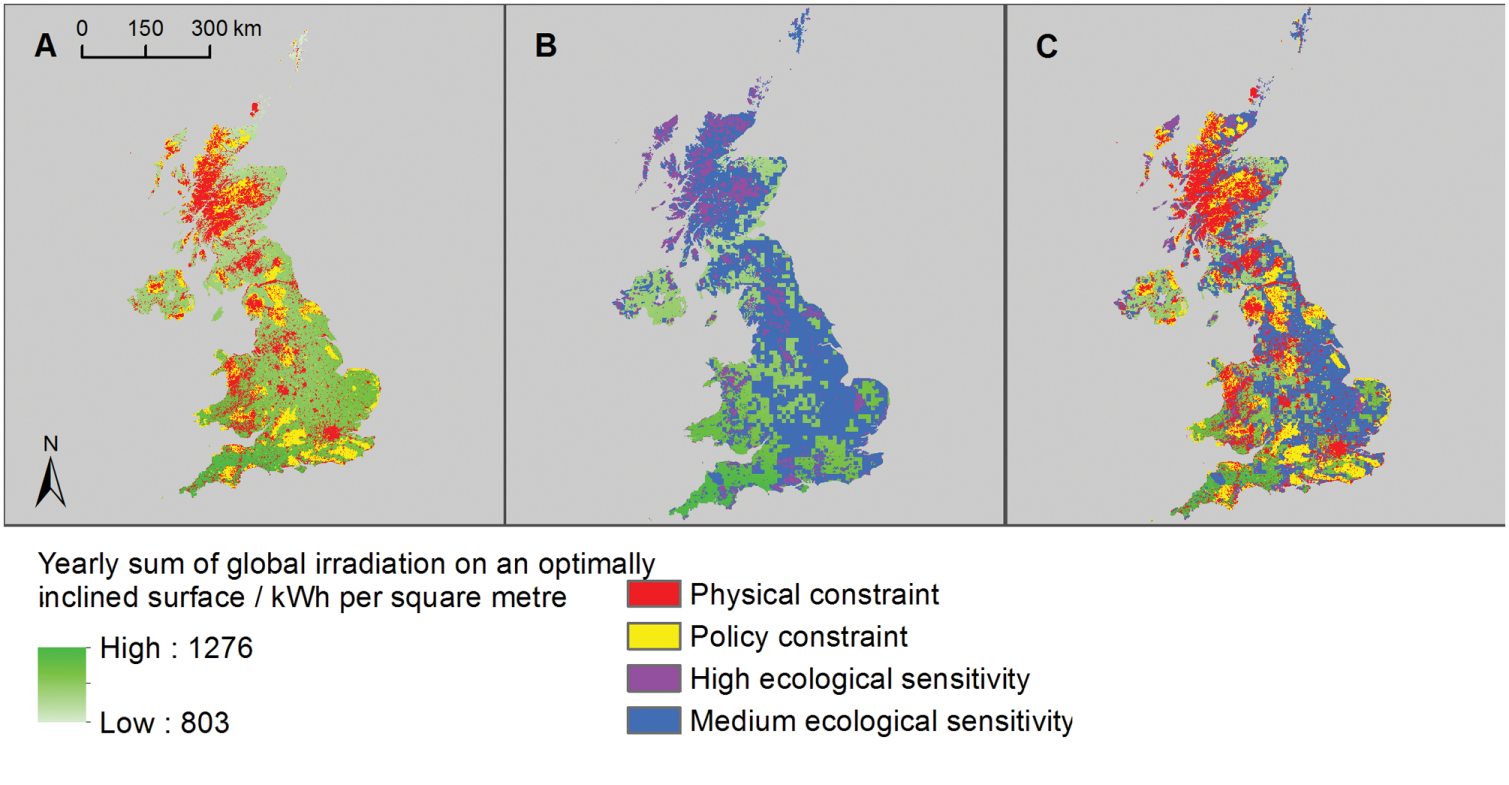
Opportunity and constraint mapping for field-scale solar energy. A) Opportunity map for solar farms in the UK (green areas) showing physical constraints (red areas) and policy constraints (yellow areas); B) ecological sensitivity map for solar farms showing high sensitivity (purple areas), medium sensitivity (blue areas) and low/unknown sensitivity (green areas); and C) composite map showing remaining areas of opportunity for solar farms with low/unknown ecological sensitivity after all constraints have been applied (green areas).

As the entire surface of the UK is exposed to sunlight, almost all of the UK land area could generate solar energy, excluding certain water bodies (S13 Table). The addition of physical and policy constraints reduced this potential to 59.8% of UK land area (Fig 5A, Table 1). The areas of sensitivity are the same as for energy crop cultivation (see Figs 3 and 5B). In the low ecological risk scenario 56,808 km^2^ (23.3% of the UK land area) was available for the deployment of solar energy (Fig 5C, Table 1). Under the medium ecological risk scenario 134,793 km^2^ (55.2% of the UK land area) was left available for solar deployment.

For solar farms, a generic approach was adopted to calculate the energy potentially available through the deployment of solar farms using an estimated power density of 22 MW/km^2^ [9]. We used an average energy output for solar farms per km^2^, as we were unable to establish a reliable methodology for estimating installed capacity from irradiance levels. To estimate annual energy output, the mean measured value for all solar farms in the UK for 2011–2013, 0.09, was used as the load factor based on average beginning and end of year capacity [37, 38]. The resultant potential annual energy output for solar farms was 986 TWh/yr for the low ecological risk scenario and 2,340 TWh/yr for the medium ecological risk scenario (Table 1).

### Offshore wind

**Fig 6:**
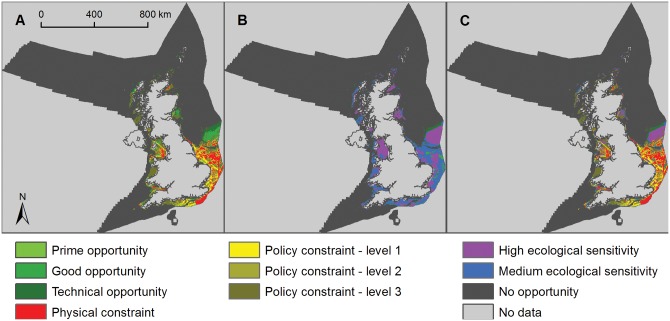
Opportunity and constraint mapping for fixed-base offshore wind energy. A) Opportunity map for fixed-base offshore wind developments (light green is prime opportunity, mid-green is good opportunity and dark green is technical opportunity showing physical constraints (red areas) and policy constraints (from level 1, least constrained, in light yellow, to level 3, most constrained, in brown); B) ecological sensitivity map for offshore wind energy showing high sensitivity (purple areas), medium sensitivity (blue areas) and low/unknown sensitivity (green areas); and C) composite map showing remaining areas of opportunity for fixed-base offshore wind turbines with low/unknown ecological sensitivity after all constraints have been applied (green areas).

**Fig 7:**
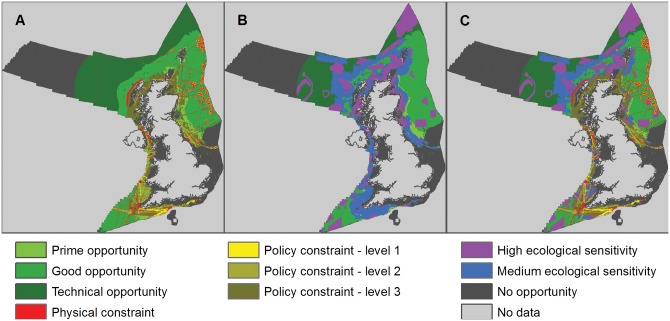
Opportunity and constraint mapping for floating offshore wind energy. A) Opportunity map for floating offshore wind developments (light green is prime opportunity, mid-green is good opportunity and dark green is technical opportunity showing physical constraints (red areas) and policy constraints (from level 1, least constrained, in light yellow, to level 3, most constrained, in brown); B) ecological sensitivity map for offshore wind energy showing high sensitivity (purple areas), medium sensitivity (blue areas) and low/unknown sensitivity (green areas); and C) composite map showing remaining areas of opportunity for floating offshore wind turbines with low/unknown ecological sensitivity after all constraints have been applied (green areas).

Relatively small areas of the UK seabed remain suitable for fixed base turbines after deployment constraints have been applied (Fig 6A, Table 2, S14 and S15 Tables). In total, 26.7% of the UK sea area potentially had high ecological sensitivity to wind turbines and 25.3% potentially had medium ecological sensitivity, leaving 48.0% as low/unknown sensitivity (Figs 3, 6B and 7B).

**Table 2:** Estimated energy available through the deployment of offshore renewable energy technologies in the UK. Estimated sea areas available for the deployment of commercial-scale offshore wind, wave and tidal energy; potential installed capacity and annual energy outputs considering the available resource, physical constraints, policy constraints and ecological sensitivity.

Renewable energy technology	High ecological risk			Medium ecological risk			Low ecological risk		
	(no sensitivity applied)			(high sensitivity areas excluded)			(high and medium sensitivity areas excluded)		
	Area (km^2^) [relative to UK sea area]^a^	Potential installed capacity (GW)	Annual energy output (TWh/yr)	Area (km^2^) [relative to UK sea area]^a^	Potential installed capacity (GW)	Annual energy output (TWh/yr)	Area (km^2^) [relative to UK sea area]^a^	Potential installed capacity (GW)	Annual energy output (TWh/yr)
**Fixed-based turbines**^b^	22,700–69,237 [2.6–7,9%]	114–346	497–1,517	7,341–11,578 [0.8%-1.3%]	37–58	161–254	3,162–5,229 [0.4–0.6%]	16–26	69–115
**Floating turbines**^b^	412,552–519,446 [47.1–59.3%]	2,063–2,597	9,041–11,383	296,813–323,999 [33.9–37.0%]	1,484–1,620	6,505–7,100	230,149–253,627 [26.3–28.9%]	1,151–1,268	5,044–5,558
**Tidal stream** ^c^	774–7,871 [0.1–0.9%]	14–138	47–483	573–5,560 [0.1–0.6%]	10–97	35–341	284–2,875 [0.0–0.3%]	5–50	17–176
**Wave**^d^	159,068–219,519 [18.1–25.0%]	-	-	129,263–171,503 [14.7–19.6%]	-	-	86,726–107,067 [9.9–12.2%]	-	-

^a^ Including all areas of opportunity (prime, good and technical; see Methodology for definitions) with physical constraints and varying levels of policy constraint applied—see Methodology and S5 Table for details.

^b^ Offshore wind turbines: power density = 5 MW/km^2^ [39]; load factor = 0.5 [40].

^c^ Tidal stream: power density = 17.5 MW/km^2^ [39]; load factor = 0.4 [39].

^d^ Potential energy extractable from waves could not be calculated on a per area basis (see [39] for details).

In the low ecological risk scenario 3,162 to 5,229 km^2^ (0.4–0.6% of the UK sea area) was available for fixed-base wind turbines, depending on the level of policy constraint applied (Fig 6C). Under the medium ecological risk scenario 7,341 to 11,578 km^2^ (0.8–1.3% of the UK sea area) was available for fixed-base wind turbines, depending on the level of policy constraint applied (Table 2; S14 Table). Substantially larger areas were potentially available for floating offshore wind turbines (Fig 7A). In the low ecological risk scenario 230,149 to 253,627 km^2^ (26.3–28.9% of the UK sea area) was available for floating wind turbines, depending on the level of policy constraint applied (Fig 7C, Table 2; S14 and S16 Tables). Under the medium ecological risk scenario 296,813 to 323,999 km^2^ (33.9–37.0% of the UK sea area) was available for floating wind turbines, depending on the level of policy constraint applied.

An average power density of 5 MW/km^2^ was used in this analysis; being approximately halfway between what is practically possible (power densities of Round 1 and 2 sites in the UK are typically 7–9 MW/km^2^) and what is currently being deployed (~2 MW/km^2^ for Round 3 sites) [39]; a load factor of 0.5 representing the best performing projects currently operating in European waters [40] was used in the analysis. This load factor was deemed to be realistic as the offshore wind industry is in its relative infancy, whereas this analysis focuses on energy production up to 2050. In the low ecological risk scenario the resultant potential annual energy output for fixed-base offshore turbines was 69–115 TWh/yr, depending on the level of policy constraint applied (Table 2, S14 Table). Under the medium ecological risk scenario 161–254 TWh/yr of potential annual energy was available from fixed-base offshore turbines, depending on the level of policy constraint applied. In the low ecological risk scenario the resultant potential annual energy output for floating offshore turbines was 5,044–5,558 TWh/yr, depending on the level of policy constraint applied (Table 2, S14 and S16 Tables). Under the medium ecological risk scenario 6,505–7,100 TWh/yr of potential annual energy was available from floating offshore turbines, depending on the level of policy constraint applied.

### Tidal stream

**Fig 8:**
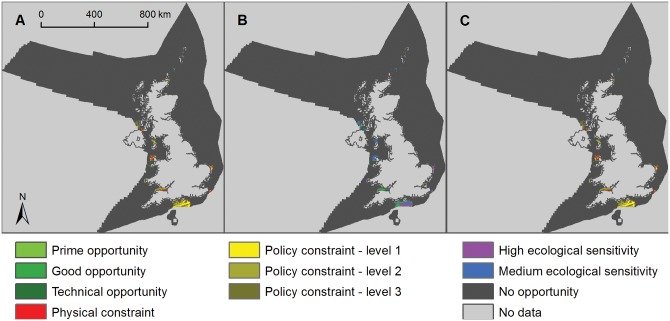
Opportunity and constraint mapping for offshore tidal stream energy. A) Opportunity map for tidal stream energy developments (light green is prime opportunity, mid-green is good opportunity and dark green is technical opportunity showing physical constraints (red areas) and policy constraints (from level 1, least constrained, in light yellow, to level 3, most constrained, in brown); B) ecological sensitivity map for tidal stream energy showing high sensitivity (purple areas), medium sensitivity (blue areas) and low/unknown sensitivity (green areas); and C) composite map showing remaining areas of opportunity for tidal stream developments with low/unknown ecological sensitivity after all constraints have been applied (green areas).

In total, 25.1% of the UK sea area potentially had high ecological sensitivity to the deployment of tidal stream technologies and 20.2% potentially had medium ecological sensitivity, leaving 54.7% with low/unknown sensitivity (Figs 3 and 8B).

Tidal stream technologies can be deployed in a limited number of locations in UK waters compared with offshore wind energy (Fig 8A). In the low ecological risk scenario 284 to 2,875 km^2^ (0.0–0.3% of the UK sea area) was available for tidal stream energy deployments, depending on the level of policy constraint applied (Fig 8C, Table 2, S14 and S17 Tables). Under the medium ecological risk scenario 573 to 5,560 km^2^ (0.1–0.6% of the UK sea area) was available for tidal stream energy, depending on the level of policy constraint applied.

The power density for tidal stream was estimated to lie in the range of 5–30 MW per km^2^ [39]. The resource size was calculated by multiplying the area practically available by the median power density estimate from this range (17.5 MW/km^2^), whilst acknowledging that this might not accurately reflect power densities at all sites. A load factor of 0.4 was used for tidal stream technologies, representing an estimate of likely future efficiencies of these technologies [39].

In the low ecological risk scenario the resultant potential annual energy output for tidal stream energy was 17–176 TWh/yr, depending on the level of policy constraint applied (Table 2, S14 and S17 Tables). Under the medium ecological risk scenario 35–341 TWh/yr of tidal stream energy was potentially available from tidal stream devices, depending on the level of policy constraint applied.

### Wave energy

**Fig 9:**
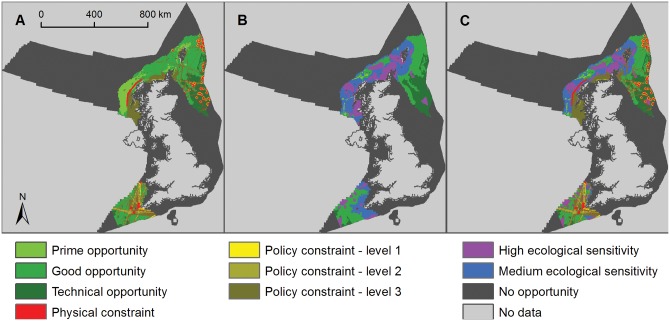
Opportunity and constraint mapping for wave energy. A) Opportunity map for wave energy developments (light green is prime opportunity, mid-green is good opportunity and dark green is technical opportunity showing physical constraints (red areas) and policy constraints (from level 1, least constrained, in light yellow, to level 3, most constrained, in brown); B) ecological sensitivity map for wave energy showing high sensitivity (purple areas), medium sensitivity (blue areas) and low/unknown sensitivity (green areas); and C) composite map showing remaining areas of opportunity for wave energy developments with low/unknown ecological sensitivity after all constraints have been applied (green areas).

A total of 26.4% of the UK sea area had high ecological sensitivity to the deployment of wave energy technologies and 22.1% had medium ecological sensitivity, leaving 51.5% of UK seas as low or unknown sensitivity (Figs 3 and 9B).

Substantial areas of sea are available for wave energy deployment (Fig 9A). In the low ecological risk scenario 86,726 to 107,067 km^2^ (9.9–12.2% of the UK sea area) was available for wave energy deployments, depending on the level of policy constraint applied (Fig 9C). Under the medium ecological risk scenario 129,263 to 171,503 km^2^ (14.7–19.6% of the UK sea area) was available for wave energy, depending on the level of policy constraint applied (Table 2; S14 and S18 Tables).

Currently, the potential energy extractable from waves cannot be calculated on a per area basis [9, 39], since a long row of efficient wave energy harvesting devices should extract a high proportion of the available energy, leaving little resource available for subsequent rows (i.e. behind the wave front). The most commonly quoted practical resource value for wave energy in UK waters is 50 TWh/yr [39]. However, conversion of 100% of the available energy is unlikely to be achievable in the near future. Indeed, Mollison [41] indicated that frequency and alignment losses may mean that only 50–70% of the practical resource is extractable energy, thus reducing the wave resource to between 25-35TWh/yr. In this analysis, we take a relatively ambitious view of the potential energy output from wave energy (35 TWh/yr), but this may need to be refined as different technologies approach market-readiness.

### Competition between onshore energy sources for land

**Fig 10:**
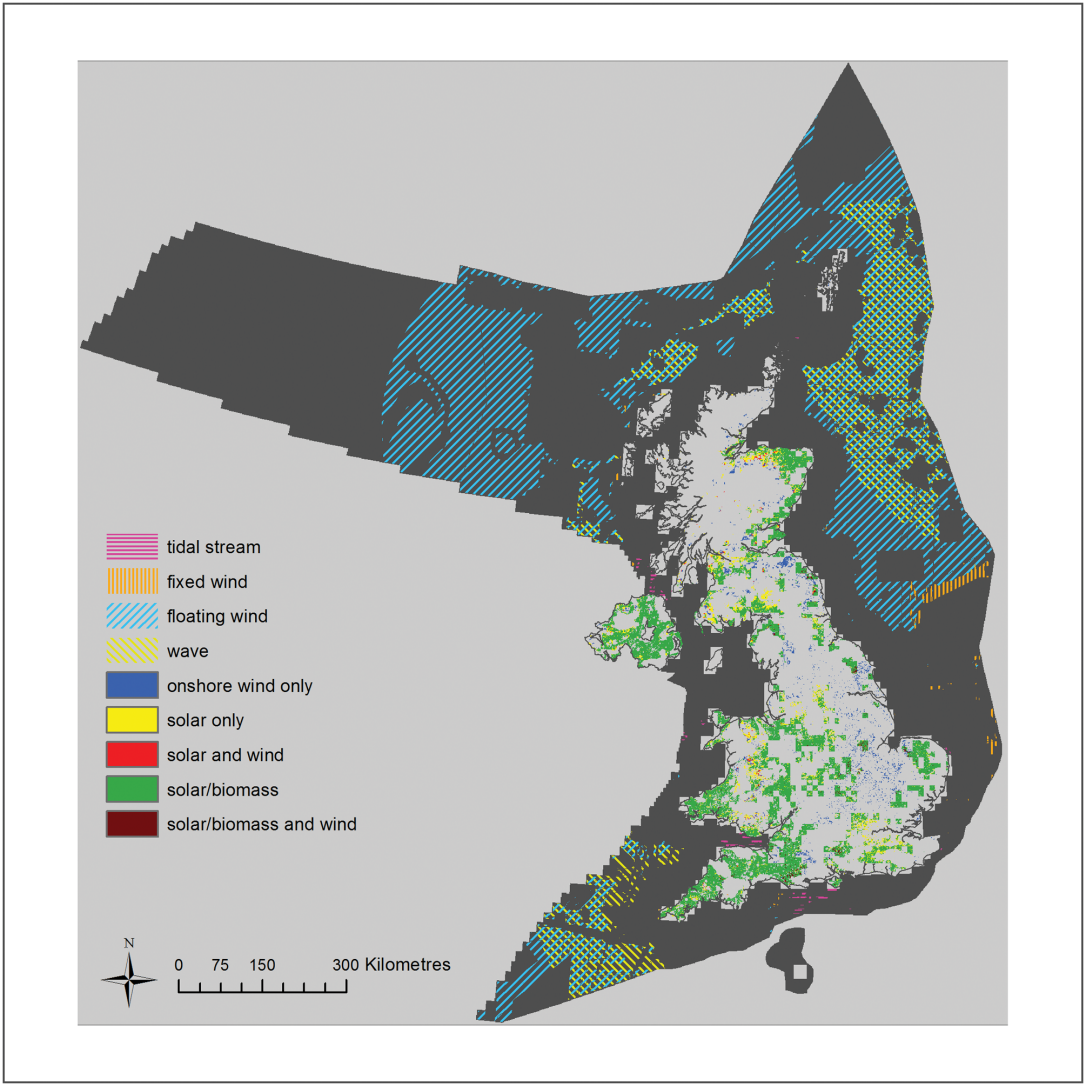
Combined map of areas of residual opportunity for renewable energy technologies in the UK. Remaining land and areas available for exploitation after all constraints (physical, policy and ecological) have been removed. Areas available for onshore wind energy are shown in blue, bioenergy or solar in green and solar only in red.

As energy crops and solar farms can be deployed in the same types of land, these energy sources are in direct competition for space (Fig 10), although solar farms can additionally be deployed in some locations that are unsuitable for energy crops. Solar farms are more efficient at producing electricity than energy crops, so it is presumed their deployment will generally be prioritised, although where there is a demand for heat as well as power this may not always be the case. In addition, it is unlikely that all of the land available for energy crops or solar farms will be utilised for energy production due to competing demands, such as the production of food/fibre and the expansion of settlements. Estimates of the amount of agricultural land available in the UK for the sustainable production of energy (bioenergy or solar) which will not displace production (resulting in indirect land-use change) vary from a conservative 3,500 km^2^ [13, 42] to more ambitious estimates e.g. 310,000 km^2^ [43] or 363,000 km^2^ [44]. If 3,500 km^2^ is the maximum amount of agricultural land available for the sustainable production of energy in the UK, and solar was prioritised over energy crops, a potential annual energy output of 61 TWh/yr could be produced. The area available for solar energy outside agricultural land (after excluding all constraints) is 10,670 km^2^, equating to an annual energy output of 185 TWh/yr. Thus, the potential annual energy output available from field-scale solar energy lies somewhere between a minimum of 246 TWh/yr (the sustainable deployment potential) and a maximum of 986 TWh/yr (the low ecological risk scenario).

### Overview of results

Looking across all of the spatially explicit (‘medium risk’) renewable energy technologies, the total potential energy output from the low ecological risk scenario was estimated to be about 5,551–6,270 TWh/yr (Table 3), whereas the medium ecological risk scenario indicates that a potential capacity of 9,325–10,319 TWh/yr exists. These figures compare favourably with future UK energy demand scenarios. For example, the current projection for 2035 is 1,442 TWh/yr [45], and for 2050, it ranges from 888 TWh/yr to 2,481 TWh/yr depending on the degree of demand reduction, energy saving and energy efficiency practiced by society [46].

**Table 3:** Summary of estimated potential energy available from ‘medium risk’ renewable energy technologies under different ecological scenarios.

Renewable energy technology	Annual Energy Output (TWh/yr)		
	High ecological risk scenario	Medium ecological risk scenario	Low ecological risk scenario
	(no sensitivity applied)	(high sensitivity areas excluded)	(medium and high sensitivity areas excluded)
**Onshore wind**	402	249	140
**Solar**^a^	2,535	2,340	246^b^
**Offshore wind (fixed)**^c^	497–1,517	161–254	69–115
**Offshore wind (floating)**^c^	9,041–11,383	6,505–7,100	5,044–5,558
**Wave energy**	35	35	35
**Tidal stream**^c^	47–483	35–341	17–176
**TOTAL**	12,557–16,355	9,325–10,319	5,551–6,270

^a^ Solar is assumed to be prioritised over bioenergy crops owing to higher rates of energy production per km^2^

^b^ Lower estimate of the range of potential sustainable energy produced from agricultural land

^c^ Figures are given for total opportunity (prime, good and technical) after physical and policy constraints (levels 1, 2 or 3) have been applied, see Methodology and S5 Table for information on components of policy levels.

## Discussion

This methodological approach has helped us understand the deployment potential of renewable energy technologies, which are likely to form a major component of future energy systems and require large areas of land or sea to be successfully deployed. The analysis revealed that the large scale deployment of renewable energy technologies could potentially contribute significantly to meeting the UK’s future energy demand, and so help commitments to reduce carbon emissions, without presenting major risks to biodiversity, even without consideration of the contribution from wider deployment of small-scale, ‘low risk’ energy technologies. It is also worth remembering that the counterfactual to the increased deployment of renewable energy, i.e. continued fossil fuel extraction and use, comes with even higher environmental costs not only from the widespread impacts of climate change [2] but also from direct impact, e.g. [47].

However, caveats remain. Firstly, it is clear that there is considerable capacity within the UK for the production of renewable energy, but further investment in research and development is necessary, particularly for offshore technologies, although considerable capacity can be deployed using existing technologies such as onshore wind and solar energy. Secondly, the ecological impacts of several renewable technologies are still poorly understood, and research and monitoring are required to reduce uncertainty about environmental impacts. While technologies such as onshore wind are already mature, widespread and with fairly well-understood impacts [3, 48–52], giving a degree of confidence in the estimated low-risk capacities, those technologies in the development phase (e.g. floating wind, wave and tidal stream) have estimated capacities that are at present largely theoretical. Whilst understanding of wildlife distributions and their sensitivities to these technologies remain partial, especially at sea, there will be a risk that renewable energy technology developments may be located inappropriately.

Thorough environmental impact assessments are necessary at all sites (not just where developments are proposed in areas identified as sensitive) to determine whether deployment at low ecological risk is feasible and to identify potential mitigation measures. The deployment of any renewable technology should be associated with a robust monitoring program to monitor sites before, during and after construction to reduce uncertainty about the impacts on biodiversity and to test potential mitigation and enhancement measures. Monitoring and research need to be targeted at those sites most likely to deliver results to improve our knowledge of specific impacts.

### Deployment constraints

The mapping has indicated that utilisation of some energy sources is limited at least as much by physical and policy constraints, as by ecological sensitivity. For example, the numerous policy constraints on the deployment of onshore wind turbines reduced the area of opportunity by 107,072 km^2^ (45.5%). The majority of this reduction is due to the 600 m buffer zone around individual houses where, at present, wind turbines are highly unlikely to get planning permission. We have assumed that policy constraints (both on and offshore) remain in place through to 2050. However, strategic priorities could change, and considerable flexibility might exist in some of the apparent constraints. If, for example, public opinion were to become more favourable towards onshore wind and this was reflected in planning guidance, areas of opportunity could be substantially larger. Consequently, large areas of land or sea that have been excluded here, may become available if the technologies develop in such a way that allows the expansion of utilisable areas, if physical constraints are removed, or if there is sufficient will exerted through policy decisions to prioritise energy production over other uses.

In this analysis it is assumed that solar farms will be deployed in preference to bioenergy crops as they produce energy more efficiently. In reality, both are likely to be deployed, since some sites might not gain planning permission for a solar farm, or a land owner may choose to prioritise bioenergy crops for economic reasons (e.g. cost of connecting to the grid, for domestic use, or to provide low carbon heat and/or transport fuels). Field-scale solar in the UK is currently concentrated in southern England, where there are higher levels of irradiance and solar farms will produce more energy per km^2^, although further development of the technology may change this situation. Therefore, solar farms in some areas may be less economically viable, especially those in the north of the UK. However, the establishment of solar farms in Aberdeenshire (Scotland) suggests that irradiance levels are not a hard constraint to deployment.

The renewable energy technologies all share the requirement for space (land or sea) for their deployment. Some are in direct competition (e.g. solar and biomass) whereas other could potentially occupy the same areas in ‘renewable energy parks’. For example there was considerable overlap between offshore wind, wave and tidal stream technologies within the opportunity mapping. As a rule we have not considered technologies to occupy areas exclusively, aside from bioenergy crops and solar farms, which are in competition for the same resource (namely sunlight). While current practice is to give consents in separate areas, it is by no means certain that this situation will continue into the future, when co-deployment might become commonplace.

However, cumulative impacts are difficult to predict, and it might be that the deployment of several of these technologies becomes constrained as the understanding of ecological consequences grows. There may also be unforeseen problems with deploying offshore technologies within ‘energy parks’, such that different offshore technologies may end up competing for the same sea areas. A certain level of deployment is likely to be possible within areas currently marked as medium (or even high) sensitivity without incurring impacts. However, a large component of this potential capacity is made up of (i) offshore floating wind turbines, which are as yet not commercially deployed, and (ii) field-scale solar farms, which would have land-use/food/fibre production consequences if widely deployed. In addition, these estimates are based on the assumption that there is suitable accessibility in terms of existing infrastructure (roads, ports etc.) to develop the areas identified and that sufficient connectivity to the grid exists; it is acknowledged that there may be other factors (cost in particular, but not alone) not included in the model that could preclude deployment.

### Onshore sensitivity mapping

The species assemblage approach we used for bioenergy crops and solar farms led to a relatively high percentage of land being classified as medium sensitivity. In large part this is due to insufficient knowledge of the potential ecological impacts of energy crop cultivation or solar farms to be able to confidently predict those species that will be most sensitive to their deployment, and the extent of potential impacts. Also the use of presence/absence data does not allow the discrimination of relative importance of specific areas or how they are used, so it is difficult to know how well sensitivity is represented.

Little research has been conducted to date on the impacts of landscape scale deployment of solar farms or bioenergy crops, for which there may be unforeseen impacts (both direct and indirect). For bioenergy in this analysis we have focused primarily on *Miscanthus* planting, as this is the most widespread biomass crop in the UK. However, other bioenergy feedstocks are also becoming more common, such as annual grass crops (e.g. maize) for biogas production. Despite this, the sensitivity map is likely to give a fairly good indication of sensitivity regardless of the nature of the crop, although crop-specific issues such as erosion, run-off or water use were not considered within the mapping process. In general there is a lack of research into the long-term effect of energy crop production, which will need to be addressed to fully understand the ecological impacts.

Although most agricultural land technically may be utilised for energy crop cultivation or solar farms, it is unrealistic to assume that the whole area classified as low ecological risk could be taken out of agricultural production, without having serious implications for food/fibre production and might potentially result in unacceptable levels of indirect land use change (ILUC) [53]. We therefore assumed that the minimum estimate of agricultural land available for energy production is 3,500 km^2^ [13, 42]. Realistically, more land could be available without ILUC issues or conflict with food/fibre production, although where the limit of sustainability lies remains unclear.

### Offshore sensitivity mapping

Aside from seabirds, the main species-specific data used in the sensitivity maps were obtained from the APEI dataset [31]. This data layer represents density/species diversity rather than sensitivity to a particular technology. As a precautionary approach, this layer was allocated a medium sensitivity score. There are information gaps with respect to the nature of impacts, the identity of sensitive species and the quality of distribution data particularly for species at-sea. Whilst the body of data has been added to considerably in recent years (not least by offshore wind farm developers), data for pelagic areas remain limited and caution must be applied when interpreting the sensitivity of areas far from the coast. Data are also limited for seabirds at breeding colonies; the last national seabird census took place between 1998 and 2002 [54], and so may not reflect current abundances. There is an urgent need to collect more at-sea data and to update the breeding seabird data with a new national seabird census. Furthermore, the network of protected sites at sea is much less comprehensive than that on land. We have partially accounted for this in the sensitivity maps by including proposed protected areas and increasing the sensitivity ratings of other sites (e.g. Important Bird Areas). However, until there is a robust network of marine protected sites, the status/importance of areas of sea with low/unknown sensitivity must be approached with caution. Whilst the mapping indicated that there were significant areas of sea available for the deployment of offshore wind energy, particularly floating turbines, it should be remembered that data on the distribution and abundance of seabirds at sea remain patchy and incomplete, becoming increasingly so further away from the shore, and the routes of migrating birds and sensitivities to potential impacts generally are poorly understood.

### Uncertainty in the mapping process and use of the sensitivity maps

The opportunity maps created for this paper were not specifically designed to identify areas that should or should not be used to deliver renewable energy, but rather to indicate the aggregated land or sea areas potentially available for renewable energy production at different levels of ecological sensitivity. Whilst the maps may be used to help inform strategic decisions, they are not of sufficiently high spatial resolution to be used for site selection purposes. The maps show the maximum areas available for technology deployment based on estimated ecological risk, and do not account for the likelihood of a technology being deployed in any specific location. There are many considerations not taken into account, for instance the economics of deployment (such as land rental prices, access, the existence of suitable infrastructure or the cost of grid connections). The analysis of the cost and likelihood of deployment are both beyond the scope of this paper. Additionally, there are some ecological risks associated with deployment that are difficult to predict and map, for example, connection to the grid and the construction of new power lines/electricity cables and cumulative impacts. The sensitivity maps we have created (or updated) as part of this analysis could be developed further (for example by addressing some of the uncertainty outlined above) and may be of strategic use when considering the expansion of renewables, both onshore and offshore, in the UK.

The implications of applying sensitivity mapping to limited and potentially incomplete lists of species requires further consideration, since it is not possible to distinguish between the low/unknown ecological sensitivity categories in the mapping process. Thus areas marked in this way should not be considered to be altogether without risk. Conversely, areas marked as sensitive are not necessarily exclusion zones with respect to renewable technologies.

### Impact mitigation and enhancement

The mitigation hierarchy (avoid-mitigate-offset) should be followed when developing plans to deploy renewables, with the main emphasis on siting installations in areas of low ecological risk. However, mitigation (and enhancement) can be used where appropriate. For example, site location, design and construction can reduce impacts on wildlife, and land may be managed to minimise interactions between renewables infrastructure and wildlife. In many locations, biodiversity gains may be possible within renewable developments, such as between or beneath solar panels, within or around bioenergy crops [26, 55] or around wind farm infrastructure [48]. The development of offshore renewables may provide shelter and substrate for diverse species, and *de facto* no-take zones around these structures are likely to enhance fish populations. Examples of management measures that might be successful include grass strips for butterflies or bees [56, 57], pollen and nectar strips [57] or winter food for birds [58]. However, the effectiveness of these measures in the context of renewable developments should be examined experimentally and thoroughly monitored, as they are currently largely untested in these environments.

### The role of technological innovation

It is very difficult to represent significant technology development within these models, particularly for a time horizon up to 2050 that could see rapid changes in the technologies available for deployment. There may be large changes to the modelling parameters that predict where the technologies can be deployed cost effectively over the coming years. None of the offshore technologies analysed in this paper, except fixed-base offshore wind, are currently available on a commercial scale. If these technologies are not progressed through to the market place, it is likely to increase the pressure to deploy those technologies that are market ready (onshore wind, solar, bioenergy and fixed-base offshore wind) in order to meet carbon emissions reduction targets. This will mean the upper estimates for their deployment potentials outlined here could potentially become a reality, increasing the risk of conflict with biodiversity. This emphasises that the modelling is a ‘snapshot’ based on current understanding of potential for technology deployment in 2015, but with an eye to the longer time-frame (2050).

It is clear that the area of deployment is not likely to be a major consideration if wave devices are deployed as linear features. However, many designs have been proposed and some of these might be deployed in more conventional array formations. It is difficult to estimate how much of the resource would really be available, since the sea required for such devices could be relatively small, but the linear front required would be very long (hundreds of kilometres).

### Contributions of other renewable technologies

We set out to understand the potential contribution that ‘medium risk’ renewable technologies could make to meeting future energy demands with different levels of estimated ecological risk. The results suggest that they can contribute significantly, albeit with some caveats, as highlighted above. But, to fully appraise how to achieve the UK’s energy future with minimal impact to biodiversity, we would also need to analyse other parts of the energy system, including supply technologies not considered in this analysis, energy demand and energy efficiency. Other renewable energy technologies with low ecological risk (such as roof-top solar, solar thermal, geothermal and heat-pumps) could be widely deployed, particularly in the built environment. Recently, for example, Hernandez et al. [59] found that solar in the built environment could produce five times the amount of energy needed to meet California’s energy demands. Estimates from the UK suggest that these technologies could make valuable contributions, given the right policy environment and incentives [9].

Renewable energy technologies are frequently criticised due to perceived risks around intermittency and security of supply. However, a wealth of options exist to deal with these issues, including diversification of sources, storage facilities, imports (of low-carbon electricity), carbon capture and storage (CCS), nuclear energy, and in certain circumstances gas (with or without CCS). Scale of markets is important too—for example, across large regions intermittency becomes of less concern (the wind is always blowing somewhere). Ecological consequences of whole energy systems are beyond the scope of this study, but should be included in the development of an energy strategy that meets carbon reduction targets with minimal effects on the natural environment.

## Conclusions

The analysis has identified that reliance only on those technologies that are currently market ready for widespread deployment (onshore wind, solar and fixed-base offshore turbines) would result in a low ecological risk deployment potential of just 455–501 TWh/yr, if the minimum estimate for land available for solar energy is used. If the upper limit for solar energy were used instead, the potential could be increased to 1,195–1,223 TWh/yr. Annual energy output under the medium ecological risk scenario could be much larger, but would undoubtedly result in ecological impacts. These outputs could make a substantial contribution towards future energy demand, which ranges from 888 TWh/yr to 2,481 TWh/yr [46], but may not be sufficient without substantial effort also being made to conserve energy and reduce demand. Novel offshore technologies (and/or expanding the envelope for fixed-base turbines) appear to be vital if the UK is to meet emissions targets by 2050 with minimal impact on biodiversity, providing that they are relatively benign ecologically. Floating offshore wind, in particular, could have the largest impact on the UK’s energy future, given the scale of the resource available and the large areas of opportunity with apparently low (or unknown) sensitivity. However, due to the relative infancy of these offshore technologies, research and development on them needs to be coupled with the continued development of an extensive offshore monitoring programme, to assess their potential impact on the UK’s marine environment and biodiversity. It should also be emphasised that if they are appropriately sited, and mitigation and enhancement measures are properly employed, the deployment of renewables offer considerable opportunities for biodiversity gains.

## Supporting Information

S1 Table Physical and policy constraints for all onshore renewable technologies. Full list of constraints, buffer distances and data sources for onshore wind, bioenergy crops and solar farms.
(PDF)

S2 Table Additional physical and policy constraints for onshore wind turbines. Constraint types with justification, associated buffer distances and data sources.
(PDF)

S3 Table Parameters used to create the opportunity maps for offshore technologies. (PDF)

S4 Table Physical constraints for all offshore technologies. Details of constraint types, associated buffer distances and data sources.
(PDF)

S5 Table Policy constraints for all offshore technologies. Including constraint types showing the technologies they are applied to and the level of constraint, from level one (least constrained) to level three (most constrained); plus data sources.
(PDF)

S6 Table Designated sites and key habitats included in the sensitivity maps. Site/habitat descriptions, their sensitivity score, indication of the technologies they applied to and data sources.
(PDF)

S7 Table Individual species included in the onshore wind sensitivity map. Details of individual species data used, resolution, buffer distances, sensitivity and data sources.
(PDF)

S8 Table Individual species included in the energy crops and solar farm sensitivity maps. Including details of buffer distances, sensitivity levels and data sources
(PDF)

S9 Table Species included at the assemblage level in the energy crops and solar farm sensitivity maps. (PDF)

S10 Table Species included in the offshore sensitivity mapping. Information sensitivity to offshore renewable technologies and foraging ranges for all seabird species commonly breeding in the UK. Two species, European storm-petrel *Hydrobates pelagicus* and Great black-backed gull *Larus marinus*, had to be excluded from the analysis due to lack of published foraging ranges.
(PDF)

S11 Table Sensitivity categories for offshore renewable energy technologies. Thresholds set using Jenks natural breaks optimisation to categorise low/unknown, medium and high sensitivity to wave power, tidal stream, wind turbine collision and displacement for breeding seabirds.
(PDF)

S12 Table Contents of the Additional Pelagic Ecological Importance (APEI) date layer. Information on the constituent datasets, processing and data sources. Areas of importance were extracted and combined to give a single polygon of high, medium and low importance for each species/group based on the classification process detailed. Data relating to seabird foraging radii were removed from the layer prior to use.
(PDF)

S13 Table Estimated energy available through the deployment of onshore renewable energy technologies in the UK. Land areas available for the deployment of commercial-scale onshore wind, solar and biomass energy; potential installed capacity and annual energy outputs considering the available resource, physical constraints, policy constraints and ecological sensitivity.
(PDF)

S14 Table Estimated energy available through the deployment of offshore renewable energy technologies in the UK. Estimated sea areas available for the deployment of commercial-scale offshore wind, wave and tidal energy; potential installed capacity and annual energy outputs considering the available resource, physical constraints, policy constraints and ecological sensitivity.
(PDF)

S15 Table Estimated energy availability through the deployment of fixed-base offshore wind energy. Areas available, potential installed capacities and annual energy output under different scenarios, considering the available resource along with physical, policy and ecological constraints.
(PDF)

S16 Table Estimated energy availability through the deployment of floating offshore wind energy. Areas available, potential installed capacities and annual energy output under different scenarios, considering the available resource along with physical, policy and ecological constraints.
(PDF)

S17 Table Estimated energy availability through the deployment of offshore tidal stream energy. Areas available, potential installed capacities and annual energy output under different scenarios, considering the available resource along with physical, policy and ecological constraints.
(PDF)

S18 Table Estimated sea areas available for the deployment of wave energy. Areas available under different scenarios, considering the available resource along with physical, policy and ecological constraints.
(PDF)
