# Supplementary material for: Reconciling Biodiversity Conservation and Widespread Deployment of Renewable Energy Technologies in the UK
Source: PLoS One. 2016 May 25;11(5):e0150956. doi: 10.1371/journal.pone.0150956 (PMC4880438; doi:10.1371/journal.pone.0150956)
Supplement: S1 Table — Full list of constraints, buffer distances and data sources for onshore wind, bioenergy crops and solar farms. (PDF) [file pone.0150956.s001.pdf]

S1 Table. Physical and policy constraints for all onshore renewable technologies. Full list of constraints, buffer distances and data sources for onshore wind, bioenergy crops and solar farms.

Constraint*	Onshore wind buffer (m)	Solar and biomass buffer (m)	Constraint type	Source
Motorways	165	15	Physical	OS Meridian2; OSNI vector 1:50000
A - roads	160	10	Physical	OS Meridian2; OSNI vector 1:50000
B - roads	155	5	Physical	OS Meridian2; OSNI vector 1:50000
Railways	157.5	7.5	Physical	OS Meridian2; OSNI vector 1:50000
Rivers and canals	7.5	7.5	Physical	OS Meridian2; OSNI vector 1:50000
Lakes and reservoirs	None	None	Physical	OS Meridian2; OSNI vector 1:50000
Settlements	600	None	Physical	OS Meridian2; OSNI vector 1:50000
Civil airports/airfields	5,000	500	Physical	CAA
Military airports/airfields	5,000	500	Physical	CAA
Scheduled monuments	None	None	Physical	Cadw; HS; NE; NIEA
World Heritage sites	None	None	Physical	Cadw; HS; NE; UNEP-WCMC
Listed buildings[a]	10	10	Physical	Cadw; HS; NE; NIEA
Ministry of Defence land[b]	None	None	Policy	MOD
Registered parks and gardens[a]	None	None	Policy	Cadw; HS; MAGIC; NIEA
Registered battlefields[a]	None	None	Policy	HS; MAGIC
National Parks[c]	None	None	Policy	Cadw; NE; NIEA; SNH
AONBs/NSAs[c]	None	None	Policy	Cadw; NE; NIEA; SNH
Wildland (Scotland)[d]	None	None	Policy	SNH

Abbreviations: AONB – Areas of Outstanding Natural Beauty; CAA – Civil Aviation Authority; Cadw is Welsh Government's historic environment service; HS – Historic Scotland; MAGIC - website provides geographic information about the natural environment (http://www.magic.gov.uk/); MOD - Ministry of Defence; NE – Natural England; NIEA Northern Ireland Environment Agency; NSA – National Scenic Areas; OS – Ordinance Survey; OSNI - Ordnance Survey of Northern Ireland; SNH- Scottish Natural Heritage; UNEP-WCMC - United Nations Environment Programme - World Conservation Monitoring Centre

*Some additional constraints were applied only to onshore wind (S3 Table). One additional physical constraint to the deployment of bioenergy crops was applied (>15% slope) since this land is difficult to plant and harvest [3].

[a]The category 'sites of historic interest' was split, with built structures (Listed buildings) categorised as physical constraints and areas of land as policy constraints.

[b]Ministry of Defence (MOD) training sites and Danger Areas were considered to be physical constraints by SQWenergy [1], but data were only available on MOD land ownership, so this larger area was included as a single overall policy constraint.

[c]National Parks and Areas of Outstanding Natural Beauty (AONBs)/National Scenic Areas (NSAs) were considered to align more closely with the policy constraint than physical constraints [1], although planning policy in Scotland has ruled these sites out for wind development at present.

[d]Areas of 'wild land' in Scotland have been recognised as 'areas of significant protection' in Scottish Planning Policy [2]. Again, this is not an absolute constraint but means more detailed assessments are likely to be required. Therefore, these have been included as policy constraints.

[1] SQWenergy. Renewable and Low-carbon Energy Capacity Methodology: Methodology for the English Regions. London: DECC & CLG; 2010. Available: https://www.gov.uk/government/uploads/system/uploads/attachment_data/file/226175/renewable_and_low_carbon_energy_capacity_methodology_jan2010.pdf. Accessed 2015 Oct 28.

[2] Scottish Government. Scottish Planning Policy. Edinburgh: Scottish Government; 2014. ISBN: 978-1-78412-567-7. Available: http://www.gov.scot/Resource/0045/00453827.pdf. Accessed 2015 Nov 04.

[3] Lovett AA, Sunnenberg GM, Richter GM, Dailey AG, Riche AB, Karp A. Land use implications of increased biomass production identified by GIS-based suitability and yield mapping for *Miscanthus* in England. Bioenergy Res 2009; 2: 17-28.
