# Supplementary material for: Reconciling Biodiversity Conservation and Widespread Deployment of Renewable Energy Technologies in the UK
Source: PLoS One. 2016 May 25;11(5):e0150956. doi: 10.1371/journal.pone.0150956 (PMC4880438; doi:10.1371/journal.pone.0150956)
Supplement: S2 Table — Constraint types with justification, associated buffer distances and data sources. (PDF) [file pone.0150956.s002.pdf]

**S2 Table. Additional physical and policy constraints for onshore wind turbines**. Constraint types with justification, associated buffer distances and data sources.

| Constraint | Buffer (km) | Constraint type | Justification | Source |
|---|---|---|---|---|
| Maximum slope angle >15° | N/A | Physical | Industry siting guidelines recommend avoiding ground steeper than 15° | OS 50 m DEM (for GB), ASTER GDEM (resampled to 50 m resolution for NI) |
| Individual Houses | 0.6 | Physical | There is no UK planning legislation to prevent turbines being built within 600 m of individual houses; however, the likelihood of obtaining planning permission is very low. | Ecotricity |
| Met Office Radar | 5 | Physical | Wind turbines can cause complications as they appear on air defence and air traffic control radar. | MoD |
| Settlements (Scotland only) | 2 | Policy | Community separation distances of 2 km were proposed as 'areas of significant protection' in the draft Scottish Planning Policy for onshore wind [1]. | OS Meridian2 |
| MoD highest priority low fly zones | None | Policy | Whilst not strict exclusion zones, planning permission is likely to be harder to obtain in these areas. | MoD |
| Eskdalemuir seismic array | 15 | Policy | The MoD has set a 15 km consultation zone, where planning permission may be difficult to obtain. | MoD |

Abbreviations: ASTER GDEM - ASTER Global Digital Elevation Model; DEM – Digital Elevation Map; GB – Great Britain; MoD – Ministry of Defence; NI – Northern Ireland; OS – Ordinance Survey

[1] Scottish Government. Scottish Planning Policy. Edinburgh: Scottish Government; 2014. ISBN: 978-1-78412-567-7. Available: http://www.gov.scot/Resource/0045/00453827.pdf. Accessed 2015 Nov 04.
