# Supplementary material for: Reconciling Biodiversity Conservation and Widespread Deployment of Renewable Energy Technologies in the UK
Source: PLoS One. 2016 May 25;11(5):e0150956. doi: 10.1371/journal.pone.0150956 (PMC4880438; doi:10.1371/journal.pone.0150956)
Supplement: S3 Table — (PDF) [file pone.0150956.s003.pdf]

S3 Table. **Parameters used to create the opportunity maps for offshore technologies.**

**Fixed Wind**	**Floating Wind**	**Tidal Stream**	**Wave Power**
Water depth (<60 m)	Water depth (>60 m minimum)	Water depth (>5 m minimum)	Water depth (10 - 200 m)
Wind speed (>8 m/s annual average)	Wind speed (>8 m/s annual average)	Tidal power (>1.5 m/s mean spring peak current)	Wave power density (>20 kW/m mean annual power)
Distance to ports and substations (m)	Distance to ports and substations (m)	Distance to ports and substations (m)	Distance to ports and substations (m)
Distance to coastline	Distance to coastline	Seabed slope	Seabed habitat score
Wave height (yearly average)	Wave height (yearly average)	Wave height (yearly average)	Wave height (yearly average)
These criteria are used by The Crown Estate in their resource assessment procedures.			
