# Supplementary material for: Reconciling Biodiversity Conservation and Widespread Deployment of Renewable Energy Technologies in the UK
Source: PLoS One. 2016 May 25;11(5):e0150956. doi: 10.1371/journal.pone.0150956 (PMC4880438; doi:10.1371/journal.pone.0150956)
Supplement: S4 Table — Details of constraint types, associated buffer distances and data sources. (PDF) [file pone.0150956.s004.pdf]

S4 Table. Physical constraints for all offshore technologies. Details of constraint types, associated buffer distances and data sources.

**Constraint**	**Technology type**	**Buffer (km)**	**Source**[c]
Active Cables[a]	All	0.5	KIS
Pipelines[a]	All	0.5	TCE; UK DEAL
Anchorage Areas[a]	All	None	SeaZone
Disposal sites[a]	All	None	CEFAS
Dredging Applications and licences[a]	All	None	TCE
Gas Storage Leases[a]	All	None	TCE
Offshore helicopter platform safety zones[a]	All	5.6[b]	TCE
International Maritime Organisation shipping routes	All	None	Anatec
Meteorological Equipment[a]	All	0.5	TCE; UK Hydrographic Office
Munitions dumps	All	None	Royal Haskoning
Munitions disposal sites	All	None	MMO
Oil and Gas (O&G) Infrastructure[a]	All	0.5	UK DEAL
O&G Safety zones[a]	All	None	UK DEAL
O&G Wells	All	0.5	UK DEAL
Obstructions to navigation	All	0.1	SeaZone
Offshore shipping zones	All	None	SeaZone
Offshore Mines[a]	All	None	TCE
Protected and designated wrecks	All	None	MCA; TCE
World Heritage sites	All	None	Cadw; CMRC; EH
Civil airports/airfields	Fixed and floating wind	5	AAA
Military airports/airfields	Fixed and floating wind	5	CAA
Met office radar	Fixed and floating wind	5	MoD

[a] Does not account for decommissioning between now and 2050
[b] Equivalent to 3 nautical miles
[c] Abbreviations: CAA – Civil Aviation Authority; Cadw – Welsh Government's historic environment service; CEFAS – Centre for Environment, Fisheries and Aquaculture Science; CMRC – Coastal and Marine Resources Centre; EH – English Heritage; KIS – Kingfisher Information Service; MCA – Maritime and Coastguard Agency; MMO – Marine Management Organisation; MoD – Ministry of Defence; TCE – The Crown Estate; UK DEAL – now UK Oil and Gas Data.
