# Supplementary material for: Reconciling Biodiversity Conservation and Widespread Deployment of Renewable Energy Technologies in the UK
Source: PLoS One. 2016 May 25;11(5):e0150956. doi: 10.1371/journal.pone.0150956 (PMC4880438; doi:10.1371/journal.pone.0150956)
Supplement: S5 Table — Including constraint types showing the technologies they are applied to and the level of constraint, from level one (least constrained) to level three (most constrained); plus data sources. (PDF) [file pone.0150956.s005.pdf]

S5 Table. Policy constraints for all offshore technologies. Including constraint types showing which technologies they are applied to and the level of constraint, from level one (least constrained) to level three (most constrained); plus data sources.

**Constraint**	**Technology type**	**Level 1 (low)**	**Level 2 (medium)**	**Level 3 (high)**	**Source**
AONBs	All	✔	✔	✔	CCW; NE; NIEA
Bivalve Harvest areas	All	✕	✕	✔	CEFAS
Fishery Orders	All	✕	✕	✔	TCE
Helicopter safety zones	All	5.6-7.4 km	5.6-9.3 km	5.6-11.1 km	TCE
Heritage Coast	All	✔	✔	✔	CCW; NE
Inshore shipping zones	All	✕	✕	Inshore	SeaZone
MoD Practice and Exercise Areas (PEXA)	All	✕	✕	✔	SeaZone
National Parks	All	✔	✔	✔	CCW; NE; SNH
National Scenic Areas	All	✔	✔	✔	SE
Royal Yachting Association Areas	All	✕	✕	✔	RYA
Scheduled Ancient Monuments	All	✕	✕	✔	Cadw; CMRC; EH; HS
Shipping density	All	Top 5%	Top 15%	Top 25%	Anatec
MoD highest priority low fly zones	Wind	✔	✔	✔	MoD

Abbreviations: Cadw – Welsh Government's historic environment service; CCW – Countryside Commission for Wales; CEFAS – Centre for Environment, Fisheries and Aquaculture Science; CMRC – Coastal and Marine Resources Centre; EH – English Heritage; HS – Historic Scotland; MoD – Ministry of Defence; NE – Natural England; NIEA – Northern Ireland Environment Agency; SNH – Scottish Natural Heritage; TCE – The Crown Estate; SE – Scottish Executive; RYA – Royal Yachting Association
