# Supplementary material for: Reconciling Biodiversity Conservation and Widespread Deployment of Renewable Energy Technologies in the UK
Source: PLoS One. 2016 May 25;11(5):e0150956. doi: 10.1371/journal.pone.0150956 (PMC4880438; doi:10.1371/journal.pone.0150956)
Supplement: S6 Table — Site/habitat descriptions, their sensitivity score, indication of the technologies they applied to and data sources. (PDF) [file pone.0150956.s006.pdf]

S6 Table. Designated sites and key habitats included in the sensitivity maps. Site/habitat descriptions, their sensitivity score, indication of which technologies to which they apply and data sources.

Site/habitat type	Technology	Sensitivity	Source
Special Protection Areas (SPAs)	All	High	[1, 2, 3, 4]
Special Areas of Conservation (SACs)	All	High	[1, 2, 3, 4]
RAMSAR sites	All	High	[1, 2, 3, 4]
Site/Area of Special Scientific Interest (SSSIs/ASSIs)	All	High	[1, 2, 3, 4]
Marine Conservation Zones (MCZs)	All offshore	High	[1]
Marine Protected Areas (including proposed MPAs)	All offshore	High	[2]
Marine Nature Reserves (MNRs)	All offshore	High	[1, 2, 3, 4]
Possible Marine SPAs for seabirds	All offshore	High	[5]
Important Bird Areas (IBAs)	Offshore/onshore	High/medium	[6]
National Nature Reserves (NNRs)	Offshore	Medium	[1, 2, 3, 4]
Nature Improvement Areas (NIAs)	Onshore	Medium	[1]
Ancient semi-natural woodland	Onshore wind	Medium	[1, 2, 3, 4]
Bog on deep peat	Onshore wind	Medium	[7]
Semi-natural grassland	Bioenergy crops/solar farms	Medium	[7]
Organic and peat soils	Bioenergy crops/solar farms	Medium	[7]

[1] Natural England; [2] Northern Ireland Environment Agency; [3] Natural Resources Wales; [4] Scottish Natural Heritage; [5] Kober K, Wilson LJ, Black J, O'Brien S, Allen S, Win I, et al. The identification of possible marine SPAs for seabirds in the UK: The application of Stage 1.1 – 1.4 of the SPA selection guidelines. JNCC Report No 461. Peterborough: Joint Nature Conservation Committee (JNCC); 2012. Available: http://jncc.defra.gov.uk/pdf/461_final_web.pdf. Accessed 2015 Oct 28; [6] BirdLife International/RSPB; [7] Land Cover Map 2000, Centre for Ecology and Hydrology. Available: http://www.ceh.ac.uk/services/land-cover-map-2000. Accessed 12 Nov 2015.
