# Supplementary material for: Reconciling Biodiversity Conservation and Widespread Deployment of Renewable Energy Technologies in the UK
Source: PLoS One. 2016 May 25;11(5):e0150956. doi: 10.1371/journal.pone.0150956 (PMC4880438; doi:10.1371/journal.pone.0150956)
Supplement: S7 Table — Details of individual species data used, resolution, buffer distances, sensitivity and data sources. (PDF) [file pone.0150956.s007.pdf]

S7 Table. Individual species included in the onshore wind sensitivity map. Details of individual species data used, resolution, buffer distances, sensitivity and data sources.

Species	Data used	Spatial resolution (km)	Buffer (km)[a]	Sensitivity[b]	Source[c]
Common scoter *Melanitta nigra*	Possible/probable/confirmed breeding records	0.1	1	high	National Survey 2007
Capercaillie *Tetrao urogallus*	Forest blocks	forest blocks	none	high	Upper survey strata from National Survey 2009-10
Black grouse *Lyrurus tetrix*	Calling males	0.01, 0.1, 1	1.5	medium	National Survey 2005, plus various regional datasets
Red-throated diver *Gavia stellata*	Possible/probable/confirmed breeding records	0.01, 0.1, 1	1	high	National Survey 2006
Black-throated diver *Gavia arctica*	Possible/probable/confirmed breeding records	0.01, 0.1, 1	1	high	National Survey 2006, plus additional records for northern Scotland
Slavonian grebe *Podiceps auritus*	Possible/probable/confirmed breeding records	0.1	1	high	Annual survey data 2003-2012
Slavonian grebe *Podiecps auritus*	Possible/probable/confirmed breeding records	0.1	1	medium	Annual survey data 1997-2002
Great Bittern *Botaurus stellaris*	Reedbeds containing nests and/or booming males	Reedbed survey polygons	none	high	Annual monitoring of nests and booming males
White-tailed eagle *Haliaeetus albicilla*	Unknown/possible/probable/ confirmed breeding records	0.01, 0.1, 1	5	high	All data since reintroduction
Osprey *Pandion haliaetus*	Possible/probable/confirmed breeding records	0.1, 1, 2	2	high	RBBP records 2002-2011
Golden eagle *Aquila chrysaetos*	Unknown/possible/probable/ confirmed breeding records	1	2.5	high	National Survey 2003
Golden eagle *Aquila chrysaetos*	Unknown/possible/probable/ confirmed breeding records	1	2.5 to 6	medium	National Survey 2003
Golden eagle *Aquila chrysaetos*	Unknown/possible/probable/ confirmed breeding records	0.1	6	medium	National Survey 1992
Red kite (Scotland) *Milvus milvus*	Nest locations	0.01, 0.1, 1	3	high	All data since reintroduction
Red kite (Scotland) *Milvus milvus*	Roost sites	0.1, 1	5	high	Annual roost counts
Hen harrier *Circus cyaneus*	Sightings	0.01, 0.1, 1	2	high	National Survey 2010
Hen harrier *Circus cyaneus*	Sightings	0.1, 1	2	medium	National Survey 2004

Marsh harrier *Circus aeruginosus*	Possible/probable/confirmed breeding records	0.1, 1	1	high	National Survey 2005
Marsh harrier *Circus aeruginosus*	Possible/probable/confirmed breeding records	0.1, 1	1-2	medium	National Survey 2006
Montagu's harrier *Circus pygargus*	Possible/probable/confirmed breeding records	0.1, 1, 2	3	high	RBBP records 2002-2011
Honey buzzard *Pernis aprivorus*	Possible/probable/confirmed breeding records	0.1, 1, 2	3	high	RBBP records 2002-2011
Honey buzzard *Pernis aprivorus*	Possible/probable/confirmed breeding records	0.1, 1, 2	3-5	medium	RBBP records 2002-2011
Peregrine falcon *Falco peregrinus*	Unknown/possible/probable/ confirmed breeding records	0.1, 1	2	medium	National Survey 2002
Merlin *Falco columbianus*	Confirmed breeding records	0.1, 1	0.5	high	National Survey 2008
Corncrake *Crex crex*	Territory centres	0.01, 0.1, 1	0.85	high	Annual surveys 2003 onwards
Crane *Grus grus*	Possible/probable/confirmed breeding records	0.1, 1, 2	2	high	RBBP records 2002-2011
Stone curlew *Burhinus oedicnemus*	Nest locations	0.01, 0.1	1	high	Annual monitoring 2008 onwards
Golden plover *Pluvialis apricaria*	10km squares with top 20% of mean counts	10	N/A	medium	1988-91 Breeding Bird Atlas
Dunlin *Calidris alpina*	10km squares with top 20% of mean counts	10	N/A	medium	1988-91 Breeding Bird Atlas
Arctic skua *Stercorarius parasiticus*	Colonies of 10 or more birds	1	none	high	Seabird 2000 (1998-2000)
Short-eared owl *Asio flammeus*	Possible/probable/confirmed breeding records	0.1, 1, 2	0.8	high	RBBP records 2002-2011
Nightjar *Caprimulgus europaeus*	Territory centres	0.01	1	high	National Survey 2004
Nightjar *Caprimulgus europaeus*	Territory centres	0.01	1-2.5	medium	National Survey 2004
Chough *Pyrrhocorax pyrrhocorax*	Confirmed breeding records	0.1, 1	1	high	National Survey 2002, plus additional records from Northern Ireland and Cornwall

[a] For full information on what buffers represent see [1, 2].
[b] Older datasets were allocated a medium sensitivity score.
[c] All data is archived and available through the RSPB Conservation Data Management Unit (CDMU); Joint Nature Conservation Committee (JNCC); Natural England (NE); Natural Resources Wales (NRW); Northern Ireland Environment Agency (NI); SNH – Scottish Natural Heritage and the British Trust for Ornithology (BTO).

[1] Bright JA, Langston RHW, Bullman R, Evans RJ, Gardner S, Pearce-Higgins J et al. Bird sensitivity map to provide locational guidance for onshore wind farms in Scotland. Edinburgh: RSPB; 2006. Available: https://www.rspb.org.uk/Images/sensitivitymapreport_tcm9-157990.pdf. Accessed 2015 Oct 27.
[2] Bright JA, Langston RHW, Anthony S. Mapped and written guidance in relation to birds and onshore wind energy development in England. Sandy: RSPB; 2009. Available:

http://www.rspb.org.uk/Images/EnglishSensitivityMap_tcm9-237359.pdf. Accessed 2015 Oct 27.
