# Supplementary material for: Reconciling Biodiversity Conservation and Widespread Deployment of Renewable Energy Technologies in the UK
Source: PLoS One. 2016 May 25;11(5):e0150956. doi: 10.1371/journal.pone.0150956 (PMC4880438; doi:10.1371/journal.pone.0150956)
Supplement: S8 Table — Including details of buffer distances, sensitivity levels and data sources (PDF) [file pone.0150956.s008.pdf]

S8 Table. Individual species included in the energy crops and solar farm sensitivity maps. Including details of buffer distances, sensitivity levels and data sources

Species	Buffer (km)		Data Source	Reference (sensitivity)
	High sensitivity	Medium sensitivity		
Black grouse *Lyrurus tetrix*	-	1.5	National Survey 2005, plus regional datasets	[1]
Golden eagle *Aquila chrysaetos*	2.5	6	National Survey 2003	[1]
Golden eagle *Aquila chrysaetos*	-	6	National Survey 1992	[1]
Hen harrier *Circus cyaneus*	2	-	National Survey 2010	[1]
Hen harrier *Circus cyaneus*	-	2	National Survey 2004	[1]
Merlin *Falco columbianus*	0.5	-	National Survey 2008	[2]
Corncrake *Crex crex*	0.85	-	Annual surveys 2003 onwards	[1]
Stone curlew *Burhinus oedicnemus*	1	-	RSPB annual monitoring surveys	[3]
Nightjar *Caprimulgus europaeus*	1	2.5	National Survey 2004	[1]
Woodlark *Lullula arborea*	0.5	1	National Survey 2006	[4]
Chough *Pyrrhocorax pyrrhocorax*	1	-	National Survey 2002 plus additional data from NI and Cornwall	[1]
Dartford warbler *Sylvia undata*	0.5	1	National Survey 2006	[4]
Cirl bunting *Emberiza cirlus*	0.5	1	National Survey 2009	[5]

[1] Bright JA, Langston RHW, Bullman R, Evans RJ, Gardner S, Pearce-Higgins J et al. Bird sensitivity map to provide locational guidance for onshore wind farms in Scotland. Edinburgh: RSPB; 2006. Available: https://www.rspb.org.uk/Images/sensitivitymapreport_tcm9-157990.pdf. Accessed 2015 Oct 27.
[2] Ruddock M, Whitfield DP. A Review of Disturbance Distances in Selected Bird Species. A report from Banchory: Natural Research Ltd.; 2007. http://www.snh.org.uk/pdfs/strategy/renewables/BIRDSD.pdf.
[3] Bright JA, Langston RHW, Anthony S. Mapped and written guidance in relation to birds and onshore wind energy development in England. Sandy: RSPB; 2009. Available: http://www.rspb.org.uk/Images/EnglishSensitivityMap_tcm9-237359.pdf. Accessed 2015 Oct 27.
[4] Currie F, Elliott G. Forests and Birds: a guide to managing forests for rare birds. Sandy: RSPB/Forestry Authority; 1997.
[5] Stevens DK, Donald PF, Evans AD, Buckingham DL, Evans J. Territory distribution and foraging patterns of cirl buntings (*Emberiza cirlus*) breeding in the UK. Biol Conserv 2002; 107: 307-313.
