# Supplementary material for: Reconciling Biodiversity Conservation and Widespread Deployment of Renewable Energy Technologies in the UK
Source: PLoS One. 2016 May 25;11(5):e0150956. doi: 10.1371/journal.pone.0150956 (PMC4880438; doi:10.1371/journal.pone.0150956)
Supplement: S9 Table — (PDF) [file pone.0150956.s009.pdf]

S9 Table. Species included at the assemblage level in the energy crops and solar farm sensitivity maps.

**Species**[a]	**Assemblage**[b]
Grey partridge *Perdix perdix*	arable
Turtle dove *Streptopelia turtur*	arable
Sky Lark *Alauda arvensis*	arable
Yellow wagtail *Motacilla flava*	arable
Whinchat *Saxicola rubetra*	arable
Tree sparrow *Passer montanus*	arable
Linnet *Carduelis cannabina*	arable
Twite *Carduelis flavirostris*	arable
Reed bunting *Emberiza schoeniclus*	arable
Yellowhammer *Emberiza citrinella*	arable
Corn bunting *Miliaria calandra*	arable
Oystercatcher *Haematopus ostralegus*	farmland wader
Northern Lapwing *Vanellus vanellus*	farmland wader
Redshank *Tringa totanus*	farmland wader
Curlew *Numenius arquata*	farmland wader
Snipe *Gallinago gallinago*	farmland wader
Golden plover *Pluvialis apricaria*	upland wader
Dunlin *Calidris alpina*	upland wader

[a] Data was obtained from *Bird Atlas 2007–11* [1], which is a joint project between BTO, BirdWatch Ireland and the Scottish Ornithologists' Club.

[b] Areas were not categorised by land cover, so if any area had a high score based on either 'upland wader', 'farmland wader' or 'arable' assemblages then it qualified as medium sensitivity.

[1] Balmer D, Gillings S, Caffrey B, Swann R, Downie I, Fuller R. Bird Atlas 2007–11: the breeding and wintering birds of Britain and Ireland. Thetford: BTO Books; 2013. ISBN 978-1-908581-28-0.
