# Supplementary material for: Reconciling Biodiversity Conservation and Widespread Deployment of Renewable Energy Technologies in the UK
Source: PLoS One. 2016 May 25;11(5):e0150956. doi: 10.1371/journal.pone.0150956 (PMC4880438; doi:10.1371/journal.pone.0150956)
Supplement: S10 Table — Information sensitivity to offshore renewable technologies and foraging ranges for all seabird species commonly breeding in the UK. Two species, European storm-petrel Hydrobates pelagicus and Great black-backed gull Larus marinus, had to be excluded from the analysis due to lack of published foraging ranges. (PDF) [file pone.0150956.s010.pdf]

S10 Table. **Species included in the offshore sensitivity mapping.** Information sensitivity to offshore renewable technologies and foraging ranges for all seabird species commonly breeding in the UK. Two species, European storm-petrel *Hydrobates pelagicus* and Great black-backed gull *Larus marinus*, had to be excluded from the analysis due to lack of published foraging ranges.

**Species**	**Offshore wind disturbance and/or displacement sensitivity score [1]**	**Offshore wind collision impacts sensitivity score [1]**	**Wave sensitivity score [2]**	**Tidal stream sensitivity score [2]**	**Mean max foraging range (km)**
Northern fulmar *Fulmarus glacialis*	2	48	80	0.5	311.43 [3]
Manx shearwater *Puffinus puffinus*	2	0	102	1.5	196.46 [3]
Leach's storm-petrel *Oceanodroma leucorhoa*	2	85	64	0.5	91.7 [4]
Northern gannet *Morus bassanus*	3	725	136	1.4	308.36 [3]
Great cormorant *Phalacrocorax carbo*	13	103	110	7.0	31.67[3]
Shag *Phalacrocorax aristotelis*	14	150	165	9.6	16.42 [3]
Great skua *Stercorarius skua*	3	320	96	0.7	42.33 [3]
Arctic skua *Stercorarius parasiticus*	3	327	84	0.6	40 [3]
Black-headed gull *Chroicocephalus ridibundus*	5	288	60	0.6	25.5 [4]
Common gull *Larus canus*	5	598	65	0.7	50 [4]
Herring gull *Larus argentatus*	3	1306	48	0.8	61.1 [4]
Lesser black-backed gull *Larus fuscus*	3	960	64	0.7	141 [4]
Black-legged kittiwake *Rissa tridactyla*	6	523	98	0.9	65.81 [3]
Little tern *Sternula albifrons*	10	212	156	0.7	6.94 [3]
Sandwich tern *Sterna sandvicensis*	9	245	125	1.1	42.3 [3]
Common tern *Sterna hirundo*	8	229	126	0.6	33.81 [3]
Arctic tern *Sterna paradisaea*	10	198	153	1.9	12.24 [3]
Roseate tern *Sterna dougallii*	9	175	135	1.0	18.28 [3]
Atlantic puffin *Fratecula arctica*	10	27	160	3.8	62.2 [3]
Black guillemot *Cepphus grylle*	16	30	169	9.9	12 [3]
Common guillemot *Uria aalge*	14	37	176	9.0	60.61 [3]
Razorbill *Alca torda*	14	-32	192	9.6	31 [3]

Data on colony size and location was obtained from the Seabird 2000 [5] colony census.

[1] Furness RW, Wade HM, Masden EA. Assessing vulnerability of marine bird populations to offshore wind farms. J Environ Manage 2013; 119: 56-66.

[2] Furness RW, Wade HM, Robbins AMC, Masden EA. Assessing the sensitivity of seabird populations to adverse effects from tidal stream turbines and wave energy devices. ICES J Mar Sci 2012; 69(8): 1466-1479.

[3] BirdLife Seabird Wikispace (http://seabird.wikispaces.com/)

[4] Thaxter CB, Lascelles B, Sugar K, Cook ASCP, Roos S, Bolton M, et al. Seabird foraging ranges as a preliminary tool for identifying candidate Marine Protected Areas. 2012; Biol Conserv 156: 53-61.

[5] Mitchell PI, Newton SF, Ratcliffe R, Dunn TE editors. Seabird Populations of Britain and Ireland: results of the Seabird 2000 census (1998-2002). London: T and A.D. Poyser; 2004.
