# Supplementary material for: Reconciling Biodiversity Conservation and Widespread Deployment of Renewable Energy Technologies in the UK
Source: PLoS One. 2016 May 25;11(5):e0150956. doi: 10.1371/journal.pone.0150956 (PMC4880438; doi:10.1371/journal.pone.0150956)
Supplement: S11 Table — Thresholds set using Jenks natural breaks optimisation to categorise low/unknown, medium and high sensitivity to wave power, tidal stream, wind turbine collision and displacement for breeding seabirds. (PDF) [file pone.0150956.s011.pdf]

S11 Table. Sensitivity categories for offshore renewable energy technologies. Thresholds set using Jenks natural breaks optimisation [1] to categorise low/unknown, medium and high sensitivity to wave power, tidal stream, wind turbine collision and displacement for breeding seabirds.

**Technology**	**Sensitivity**	**Threshold**
Wave	Low/unknown	0 – 440
	Medium	440 – 1,461
	High	1,641 – 4,619
Tidal stream	Low/unknown	0 – 4
	Medium	4 – 8
	High	8 – 16
Wind turbines (collision)	Low/unknown	0 – 739
	Medium	739 – 2,346
	High	2,346 – 13,515
Wind turbines (displacement)	Low/unknown	0 – 34
	Medium	34 – 92
	High	92 – 184
[1] Jenks GF. The data model concept in statistical mapping. International Yearbook of Cartography 1967; 7: 186–190.		
