# Supplementary material for: Reconciling Biodiversity Conservation and Widespread Deployment of Renewable Energy Technologies in the UK
Source: PLoS One. 2016 May 25;11(5):e0150956. doi: 10.1371/journal.pone.0150956 (PMC4880438; doi:10.1371/journal.pone.0150956)
Supplement: S12 Table — Information on the constituent datasets, processing and data sources. Areas of importance were extracted and combined to give a single polygon of high, medium and low importance for each species/group based on the classification process detailed. Data relating to seabird foraging radii were removed from the layer prior to use. (PDF) [file pone.0150956.s012.pdf]

S12 Table. Contents of the Additional Pelagic Ecological Importance (APEI) date layer. Information on the constituent datasets, processing and data sources. Areas of importance were extracted and combined to give a single polygon of high, medium and low importance for each species/group based on the classification process detailed. Data relating to seabird foraging radii were removed from the layer prior to use. All parameters are scored on a scale of 0-3 except for thermal fronts which was scored from 1-3.

**Theme**	**Name**	**Data description**	**Classification**[a]	**Source**
Nursery and spawning grounds	Cefas & ICES nursery/spawning grounds (1998 improved)	Single shape files showing distribution of nursery/spawning grounds for selected fish species.	**Low importance** = presence of low importance spawning or nursery ground for any species (irrespective of how many species)<br>**Moderate importance** = presence of high importance spawning or nursery grounds for single species<br>**High importance** = presence of high importance spawning or nursery grounds for two or more species	[1]
Thermal fronts	Summer oceanic thermal fronts	Frequent front maps for the summer at 4 km resolution, representing the percentage of time a strong front was observed at each grid cell location.	**Low importance** = 0 - 50% frontal frequency<br>**Moderate importance** = 50 - 80% frontal frequency<br>**High importance** = 80 - 100% frontal frequency	[1]
Basking Shark *Cetorhinus maximus*	Sightings data	Long term sightings data from around the UK for basking sharks were transcribed into a grid data layer.	**Low importance** = 1-10 sightings<br>**Moderate importance** = 10-270 sightings<br>**High importance** = 270+ sightings	[2]
Marine mammals	Important areas for marine mammals	Polygon data for important areas for marine mammals were extracted from WDCS report.	**Low importance** = Presence of areas of interest (irrespective of how many species)<br>**Moderate importance** = Presence of critical area of importance for one species<br>**High importance** = Presence of 2 or more areas of critical importance	[3]

CEFAS – Centre for Environment, Fisheries and Aquaculture Science; ICES - International Council for the Exploration of the Sea

[a] Low importance areas are allocated a score of 1, moderate areas a score of 2 and high importance areas a score of 3.

[1] Brown C, Hull S, Warken N. Accessing and developing the required biophysical datasets and data layers for Marine Protected Areas network planning and wider marine spatial planning purposes. Report No 26: MB0102 Final Project Report. Southampton: ABP Marine Environmental Research Ltd (ABPmer); 2012.

[2] Bloomfield A, Solandt, J-L. Marine Conservation Society Basking Shark Watch 20 year report 1987-2006. Ross on Wye: Marine Conservation Society; 2007.

[3] Clark J, Dolman SJ, Hoyt E. Towards Marine Protected Areas for Cetaceans in Scotland, England and Wales: A Scientific Review Identifying Critical Habitat with Key Recommendations. Chippenham: Whale and Dolphin Conservation Society 2010.
