# Supplementary material for: Reconciling Biodiversity Conservation and Widespread Deployment of Renewable Energy Technologies in the UK
Source: PLoS One. 2016 May 25;11(5):e0150956. doi: 10.1371/journal.pone.0150956 (PMC4880438; doi:10.1371/journal.pone.0150956)
Supplement: S13 Table — Land areas available for the deployment of commercial-scale onshore wind, solar and biomass energy; potential installed capacity and annual energy outputs considering the available resource, physical constraints, policy constraints and ecological sensitivity. (PDF) [file pone.0150956.s013.pdf]

S13 Table. Estimated energy available through the deployment of onshore renewable energy technologies in the UK. Land areas available for the deployment of commercial-scale onshore wind, solar and biomass energy; potential installed capacity and annual energy outputs considering the available resource, physical constraints, policy constraints and ecological sensitivity.

Onshore technologies		High ecological risk (no sensitivity applied)			Medium ecological risk (high sensitivity areas excluded)			Low ecological risk (high and medium sensitivity areas excluded)		
**Opportunity**	**Constraints**	**Area (km^2) [relative to UK land area]**	**Potential installed capacity (GW)**	**Annual energy output (TWh/yr)**	**Area (km^2) [relative UK land area]**	**Potential installed capacity (GW)**	**Annual energy output (TWh/yr)**	**Area (km^2) [relative to UK land area]**	**Potential installed capacity (GW)**	**Annual energy output (TWh/yr)**
**Onshore wind**[a]	***None***	235,398 [96.3%]	2,119	5,571	190,581 [78.0%]	1,715	4,511	150,154 [61.5%]	1,351	3,554
	Physical only	124,048 [50.8%]	1,116	2,936	94,011 [38.5%]	846	2,225	69,967 [28.6%]	630	1,656
	Physical + Policy	16,976 [6.9%]	153	402	10,523 [4.3%]	95	249	5,932 [2.4%]	53	140
**Solar**[b]	***None***	242,605 [99.3%]	5,337	4,211	206,284 [84.4%]	4,543	3,580	81,199 [33.2%]	1,786	1,409
	Physical only	182,148 [74.5%]	4,007	3,161	161,047 [65.9]	3,543	2,795	64,513 [26.4%]	1,419	1,120
	Physical and Policy	146,028 [59.8%]	3,213	2,535	134,793 [55.2%]	2,965	2,340	56,808 [23.3%]	1,250	986
**Biomass**[c]	***None***	127,977 [52.4%]	96	445	124,302 [50.9%]	93	432	56,374 [23.1%]	42	196
	Physical only	118,744 [48.6%]	89	413	115,576 [47.3%]	87	402	51,371 [21.0]	39	179
	Physical and Policy	104,289 [42.7%]	78	362	102,058 [41.8%]	77	355	46,138 [18.9%]	35	160

[a] Onshore wind: power density = 9 MW/km^2 [1]; load factor = 0.3 [2, 3].
[b] Solar photovoltaics: power density = 22 MW/km^2 [4]; load factor = 0.09 [2, 3].
[c] Biomass electricity: power density = 0.75 MW/km^2 [4]; load factor = 0.53 [2, 3].

[1] SQWenergy. Renewable and Low-carbon Energy Capacity Methodology: Methodology for the English Regions. London: DECC & CLG; 2010. Available: https://www.gov.uk/government/uploads/system/uploads/attachment_data/file/226175/renewable_and_low_carbon_energy_capacity_methodology_jan2010.pdf.

Accessed 2015 Oct 28.
[2] DECC. Digest of United Kingdom energy statistics (DUKES) 2014. London: Department of Energy and Climate Change (DECC); 2014. Available: https://www.gov.uk/government/uploads/system/uploads/attachment_data/file/338750/DUKES_2014_printed.pdf. Accessed 2015 Oct 27.
[3] DECC. Digest of United Kingdom energy statistics (DUKES) 2015. London: Department of Energy and Climate Change (DECC); 2015. Available: https://www.gov.uk/government/uploads/system/uploads/attachment_data/file/450302/DUKES_2015.pdf. Accessed 2015 Oct 27.
[4] MacKay DJC. Sustainable Energy - without the hot air. Cambridge: UIT Cambridge Ltd.; 2009. Available: http://www.inference.eng.cam.ac.uk/sustainable/book/tex/sewtha.pdf. Accessed 2015 Oct 28.
