# Supplementary material for: Reconciling Biodiversity Conservation and Widespread Deployment of Renewable Energy Technologies in the UK
Source: PLoS One. 2016 May 25;11(5):e0150956. doi: 10.1371/journal.pone.0150956 (PMC4880438; doi:10.1371/journal.pone.0150956)
Supplement: S14 Table — Estimated sea areas available for the deployment of commercial-scale offshore wind, wave and tidal energy; potential installed capacity and annual energy outputs considering the available resource, physical constraints, policy constraints and ecological sensitivity. (PDF) [file pone.0150956.s014.pdf]

S14 Table. Estimated energy available through the deployment of offshore renewable energy technologies in the UK. Estimated sea areas available for the deployment of commercial-scale offshore wind, wave and tidal energy; potential installed capacity and annual energy outputs considering the available resource, physical constraints, policy constraints and ecological sensitivity.

Offshore technologies		High ecological risk (no ecological sensitivity applied)			Medium ecological risk (high sensitivity areas excluded)			Low ecological risk (medium and high sensitivity areas excluded)		
**Opportunity**[a]	**Constraints**	**Area (km^2) [relative to UK sea area]**	**Potential installed capacity (GW)**	**Annual energy output (TWh/yr)**	**Area (km^2) [relative to UK sea area]**	**Potential installed capacity (GW)**	**Annual energy output (TWh/yr)**	**Area (km^2) [relative to UK sea area]**	**Potential installed capacity (GW)**	**Annual energy output (TWh/yr)**
**Fixed wind turbines**[b]	***None***	106,173 [12.1%]	531	2,327	14,453 [1.6%]	72	317	7,242 [0.8%]	36	159
	Physical only	82,848 [9.5%]	414	1,816	12,499 [1.4%]	62	274	5,859 [0.7%]	29	128
	Physical + Policy level 1	69,237 [7.9%]	346	1,517	11,578 [1.3%]	58	254	5,229 [0.6%]	26	115
	Physical +Policy levels 1 & 2	51,923 [5.9%]	260	1,138	9,977 [1.1%]	50	219	4,134 [0.5%]	21	91
	Physical + Policy levels 1, 2 & 3	22,700 [2.6%]	114	497	7,341 [0.8%]	37	161	3,162 [0.4%]	16	69
**Floating turbines**[b]	***None***	561,208 [64.0%]	2,806	12,299	346,013 [39.5%]	1,730	7,583	273,024 [31.1%]	1,365	5,983
	Physical only	530,514 [60.5%]	2,653	11,626	328,628 [37.5%]	1,643	7,202	257,875 [29.4%]	1,289	5,651
	Physical and Policy level 1	519,446 [59.3%]	2,597	11,383	323,999 [37.0%]	1,620	7,100	253,627 [28.9%]	1,268	5,558
	Physical and Policy levels 1 & 2	489,519 [55.8%]	2,448	10,728	316,187 [36.1%]	1,581	6,929	247,157 [28.2%]	1,236	5,416
	Physical and Policy levels 1, 2 & 3	412,552 [47.1%]	2,063	9,041	296,813 [33.9%]	1,484	6,505	230,149 [26.3%]	1,151	5,044
**Tidal stream**[c]	***None***	12,246 [1.4%]	214	751	8,089 [0.9%]	142	496	4,291 [0.5%]	75	263
	Physical only	10,764 [1.2%]	188	660	6,939 [0.8%]	121	426	3,728 [0.4%]	65	229
	Physical and Policy level 1	7,871 [0.9%]	138	483	5,560 [0.6%]	97	341	2,875 [0.3%]	50	176
	Physical and Policy levels 1 & 2	4,633 [0.5%]	81	284	3,458 [0.4%]	61	212	1,647 [0.2%]	29	101
	Physical and Policy levels 1, 2 & 3	774 [0.1%]	14	47	573 [0.1%]	10	35	284 [0.0%]	5	17
**Wave**[d]	***None***	244,546 [27.9%]	-	-	192,594 [22.0%]	-	-	121,272 [13.8%]	-	-

	Physical only	223,705 [25.5%]	-	-	174,567 [19.9%]	-	-	109,387 [12.5%]	-	-
	Physical and Policy level 1	219,519 [25.0%]	-	-	171,503 [19.6%]	-	-	107,067 [12.2%]	-	-
	Physical and Policy levels 1 & 2	206,311 [23.5%]	-	-	159,844 [18.2%]	-	-	99,830 [11.4%]	-	-
	Physical and Policy levels 1, 2 & 3	159,068 [18.1%]	-	-	129,263 [14.7%]	-	-	86,726 [9.9%]	-	-

[a] Including all areas of opportunity (prime, good and technical, see Methodology for definitions); policy levels - see Methodology and S5 Table for details.

[b] Offshore wind turbines: power density = 5 MW/km^2 [1]; load factor = 0.5 [2].

[c] Tidal stream: power density = 17.5 MW/km^2 [1]; load factor = 0.4 [1].

[d] Potential energy extractable from waves could not be calculated on a per area basis (see [1] for details).

[1] The Offshore Valuation Group. The Offshore Valuation: A valuation of the UK's offshore renewable energy resource. Machynlleth: Public Interest Research Centre; 2010. Available: http://www.ppaenergy.co.uk/web-resources/resources/467ac5b8919.pdf. Accessed 2015 Oct 28.

[2] Energy Numbers. Capacity factors a Danish offshore wind farms. Available: http://energynumbers.info/capacity-factors-at-danish-offshore-wind-farms. Accessed 2015 Nov 04.
