# Supplementary material for: Reconciling Biodiversity Conservation and Widespread Deployment of Renewable Energy Technologies in the UK
Source: PLoS One. 2016 May 25;11(5):e0150956. doi: 10.1371/journal.pone.0150956 (PMC4880438; doi:10.1371/journal.pone.0150956)
Supplement: S15 Table — Areas available, potential installed capacities and annual energy output under different scenarios, considering the available resource along with physical, policy and ecological constraints. (PDF) [file pone.0150956.s015.pdf]

S15 Table. Estimated energy availability through the deployment of fixed-base offshore wind energy. Areas available, potential installed capacities and annual energy output under different scenarios, considering the available resource along with physical, policy and ecological constraints.

**Fixed-base offshore turbines**		**High ecological risk scenario**			**Medium ecological risk scenario**			**Low ecological risk scenario**		
		**(no sensitivity applied)**			**(high sensitivity areas excluded)**			**(medium and high sensitivity areas excluded)**		
**Opportunity**	**Constraints**	**Area (km^2)**	**Potential installed capacity (GW)**	**Annual energy output (TWh/yr)**	**Area (km^2)**	**Potential installed capacity (GW)**	**Annual energy output (TWh/yr)**	**Area (km^2)**	**Potential installed capacity (GW)**	**Annual energy output (TWh/yr)**
Prime	*None*	14,374	72	315	843	4	18	264	1	6
	Physical only	11,903	60	261	679	3	15	191	1	4
	Physical + Policy level 1	8,555	43	187	498	2	11	95	0	2
	Physical +Policy levels 1 & 2	7,149	36	157	435	2	10	60	0	1
	Physical + Policy levels 1, 2 & 3	1,536	8	34	107	1	2	37	0	1
Prime & good	*None*	89,595	448	1963	9,252	46	203	3,617	18	79
	Physical only	67,639	338	1482	7,436	37	163	2,333	12	51
	Physical + Policy level 1	54,842	274	1202	6,591	33	144	1,763	9	39
	Physical + Policy levels 1 & 2	39,296	196	861	5,190	26	114	813	4	18
	Physical + Policy levels 1, 2 & 3	15,276	76	335	3,049	15	67	233	1	5
Prime, good & technical	*None*	106,173	531	2327	14,453	72	317	7,242	36	159
	Physical only	82,848	414	1816	12,499	62	274	5,859	29	128
	Physical + Policy level 1	69,237	346	1517	11,578	58	254	5,229	26	115
	Physical + Policy levels 1 & 2	51,923	260	1138	9,977	50	219	4,134	21	91
	Physical + Policy levels 1, 2 & 3	22,700	114	497	7,341	37	161	3,162	16	69

Power density = 5 MW/km^2 [1]; load factor = 0.5 [2].

[1] The Offshore Valuation Group. The Offshore Valuation: A valuation of the UK's offshore renewable energy resource. Machynlleth: Public Interest Research Centre; 2010. Available: http://www.ppaenergy.co.uk/web-resources/resources/467ac5b8919.pdf. Accessed 2015 Oct 28.
[2] Energy Numbers. Capacity factors a Danish offshore wind farms. Available: http://energynumbers.info/capacity-factors-at-danish-offshore-wind-farms. Accessed 2015 Nov 04.
