# Supplementary material for: Reconciling Biodiversity Conservation and Widespread Deployment of Renewable Energy Technologies in the UK
Source: PLoS One. 2016 May 25;11(5):e0150956. doi: 10.1371/journal.pone.0150956 (PMC4880438; doi:10.1371/journal.pone.0150956)
Supplement: S17 Table — Areas available, potential installed capacities and annual energy output under different scenarios, considering the available resource along with physical, policy and ecological constraints. (PDF) [file pone.0150956.s017.pdf]

S17 Table. Estimated energy availability through the deployment of offshore tidal stream energy. Areas available, potential installed capacities and annual energy output under different scenarios, considering the available resource along with physical, policy and ecological constraints.

**Offshore tidal stream**		**High ecological risk scenario (no sensitivity applied)**			**Medium ecological risk scenario (high sensitivity areas excluded)**			**Low ecological risk scenario (medium and high sensitivity areas excluded)**		
**Opportunity**	**Constraints**	**Area (km^2)**	**Potential installed capacity (GW)**	**Annual energy output (TWh/yr)**	**Area (km^2)**	**Potential installed capacity (GW)**	**Annual energy output (TWh/yr)**	**Area (km^2)**	**Potential installed capacity (GW)**	**Annual energy output (TWh/yr)**
Prime	*None*	964	17	59	601	11	37	264	5	16
	Physical only	914	16	56	556	10	34	229	4	14
	Physical + Policy level 1	789	14	48	486	9	30	168	3	10
	Physical +Policy levels 1 & 2	568	10	35	393	7	24	125	2	8
	Physical + Policy levels 1, 2 & 3	47	1	3	27	0	2	7	0	0
Prime & good	*None*	10,437	183	640	6,715	118	412	3,280	57	201
	Physical only	9,376	164	575	5,921	104	363	2,979	52	183
	Physical + Policy level 1	6,747	118	414	4,656	81	286	2,219	39	136
	Physical + Policy levels 1 & 2	3,826	67	235	2,818	49	173	1,187	21	73
	Physical + Policy levels 1, 2 & 3	610	11	37	448	8	27	168	3	10
Prime, good & technical	*None*	12,246	214	751	8,089	142	496	4,291	75	263
	Physical only	10,764	188	660	6,939	121	426	3,728	65	229
	Physical + Policy level 1	7,871	138	483	5,560	97	341	2,875	50	176
	Physical + Policy levels 1 & 2	4,633	81	284	3,458	61	212	1,647	29	101
	Physical + Policy levels 1, 2 & 3	774	14	47	573	10	35	284	5	17

Power density = 17.5 MW/km^2 [1]; load factor = 0.4 [1].

[1] The Offshore Valuation Group. The Offshore Valuation: A valuation of the UK's offshore renewable energy resource. Machynlleth: Public Interest Research Centre; 2010. Available: http://www.ppaenergy.co.uk/web-resources/resources/467ac5b8919.pdf. Accessed 2015 Oct 28.
