# Supplementary material for: Reconciling Biodiversity Conservation and Widespread Deployment of Renewable Energy Technologies in the UK
Source: PLoS One. 2016 May 25;11(5):e0150956. doi: 10.1371/journal.pone.0150956 (PMC4880438; doi:10.1371/journal.pone.0150956)
Supplement: S18 Table — Areas available under different scenarios, considering the available resource along with physical, policy and ecological constraints. (PDF) [file pone.0150956.s018.pdf]

S18 Table. Estimated sea areas available for the deployment of wave energy. Areas available under different scenarios, considering the available resource along with physical, policy and ecological constraints.

**Offshore wave energy**		**High ecological risk scenario**	**Medium ecological risk scenario**	**Low ecological risk scenario**
		**(no sensitivity applied)**	**(high sensitivity areas excluded)**	**(medium and high sensitivity areas excluded)**
**Opportunity**	**Constraints**	**Area (km^2)**	**Area (km^2)**	**Area (km^2)**
Prime	*None*	34,411	23,580	2,868
	Physical only	31,918	21,672	2,832
	Physical + Policy level 1	31,708	21,608	2,829
	Physical +Policy levels 1 & 2	31,386	21,365	2,829
	Physical + Policy levels 1, 2 & 3	25,054	18,296	2,641
Prime & good	*None*	200,214	151,439	80,140
	Physical only	185,788	139,230	74,071
	Physical + Policy level 1	183,050	137,473	73,058
	Physical + Policy levels 1 & 2	171,927	127,721	67,728
	Physical + Policy levels 1, 2 & 3	129,235	101,419	58,903
Prime, good & technical	*None*	244,546	192,594	121,272
	Physical only	223,705	174,567	109,387
	Physical + Policy level 1	219,519	171,503	107,067
	Physical + Policy levels 1 & 2	206,311	159,844	99,830
	Physical + Policy levels 1, 2 & 3	159,068	129,263	86,726
