# Supplementary material for: Reconciling Biodiversity Conservation and Widespread Deployment of Renewable Energy Technologies in the UK
Source: PLoS One. 2016 May 25;11(5):e0150956. doi: 10.1371/journal.pone.0150956 (PMC4880438; doi:10.1371/journal.pone.0150956)
Supplement: S16 Table — Areas available, potential installed capacities and annual energy output under different scenarios, considering the available resource along with physical, policy and ecological constraints. (PDF) [file pone.0150956.s016.pdf]

S16 Table. Estimated energy availability through the deployment of floating offshore wind energy. Areas available, potential installed capacities and annual energy output under different scenarios, considering the available resource along with physical, policy and ecological constraints.

**Floating offshore turbines**		**High ecological risk scenario (no sensitivity applied)**			**Medium ecological risk scenario (high sensitivity areas excluded)**			**Low ecological risk scenario (medium and high sensitivity areas excluded)**		
**Opportunity**	**Constraints**	**Area (km^2)**	**Potential installed capacity (GW)**	**Annual energy output (TWh/yr)**	**Area (km^2)**	**Potential installed capacity (GW)**	**Annual energy output (TWh/yr)**	**Area (km^2)**	**Potential installed capacity (GW)**	**Annual energy output (TWh/yr)**
Prime	*None*	109,573	548	2,401	14,632	73	321	12,405	62	272
	Physical only	100,606	503	2,205	13,617	68	298	11,475	57	251
	Physical + Policy level 1	94,029	470	2,061	13,003	65	285	10,945	55	240
	Physical +Policy levels 1 & 2	74,409	372	1,631	12,136	61	266	10,300	52	226
	Physical + Policy levels 1, 2 & 3	26,619	133	583	8,152	41	179	7,241	36	159
Prime & good	*None*	365,996	1,830	8,021	198,851	994	4,358	171,088	855	3,749
	Physical only	337,193	1,686	7,389	182,701	914	4,004	156,724	784	3,435
	Physical + Policy level 1	326,125	1,631	7,147	178,072	890	3,902	152,476	762	3,341
	Physical + Policy levels 1 & 2	296,198	1,481	6,491	170,260	851	3,731	146,006	730	3,200
	Physical + Policy levels 1, 2 & 3	219,233	1,096	4,804	150,886	754	3,307	128,998	645	2,827
Prime, good & technical	*None*	561,208	2,806	12,299	346,013	1,730	7,583	273,024	1,365	5,983
	Physical only	530,514	2,653	11,626	328,628	1,643	7,202	257,875	1,289	5,651
	Physical + Policy level 1	519,446	2,597	11,383	323,999	1,620	7,100	253,627	1,268	5,558
	Physical + Policy levels 1 & 2	489,519	2,448	10,728	316,187	1,581	6,929	247,157	1,236	5,416
	Physical + Policy levels 1, 2 & 3	412,552	2,063	9,041	296,813	1,484	6,505	230,149	1,151	5,044

Power density = 5 MW/km^2 [1]; load factor = 0.5 [2].

[1] The Offshore Valuation Group. The Offshore Valuation: A valuation of the UK's offshore renewable energy resource. Machynlleth: Public Interest Research Centre; 2010. Available: http://www.ppaenergy.co.uk/web-resources/resources/467ac5b8919.pdf. Accessed 2015 Oct 28.
[2] Energy Numbers. Capacity factors a Danish offshore wind farms. Available: http://energynumbers.info/capacity-factors-at-danish-offshore-wind-farms. Accessed 2015 Nov 04.
